# Patient safety incidents in the psychiatric inpatient setting: determinants, consequences, and strategies. A systematic review

**DOI:** 10.3389/fpsyt.2025.1703768

**Published:** 2026-01-14

**Authors:** Sophia Russotto, Andrea Conti, Alice Masini, Silvia Tempia Valenta, Kris Vanhaecht, José Joaquin Mira, Massimiliano Panella

**Affiliations:** 1 Department of Translational Medicine, Università del Piemonte Orientale, Novara, Italy; 2 Doctoral Program in Sports and Health—Patient Safety Line, Universitas Miguel Hernandez, Alicante, Spain; 3 Food, Health, and Longevity Doctoral Program, Università del Piemonte Orientale, Novara, Italy; 4 Department of Biomedical and Neuromotor Sciences, University of Bologna, Bologna, Italy; 5 Doctoral Program of Global Health, Humanitarian Aid and Disaster Medicine, Vrije Universiteit, Brussel, Belgium; 6 Leuven Institute for Healthcare Policy, KU Leuven, Leuven, Belgium; 7 Atenea Research, FISABIO, Hermanos López de Osaba, Alicante, Spain; 8 Direzione Medica dei Presidi Ospedalieri, Azienda Ospedaliero-Universitaria di Alessandria, Alessandria, Italy

**Keywords:** accidents, patient harm, patient safety, psychiatry, public health, safety

## Abstract

**Introduction:**

Patient safety in psychiatric inpatient settings remains an underexplored area despite the heightened vulnerability of this population to preventable harm. This review aimed to provide an updated and comprehensive overview of Patient Safety Incidents (PSIs) in psychiatric inpatient settings, identifying their types, contributing factors, preventive strategies, consequences, and mitigating actions.

**Methods:**

A systematic search was conducted in PubMed, Embase and Scopus for primary studies published from 2000 onward. A total of 92 studies were included. Data were synthesized using the World Health Organization’s International Classification for Patient Safety as the guiding framework.

**Results:**

The most frequently reported PSIs included behavior-related incidents (self-harm, suicide attempts, and patient aggression), medication-related events, and patient falls. Contributing factors were predominantly linked to patient characteristics (e.g., psychiatric symptoms), staff performance and communication issues, organizational shortcomings (e.g., inadequate protocols), and environmental hazards (e.g., unsafe physical infrastructure). Preventive actions primarily focused on improving safety culture, staff training, and environmental modifications. However, only a minority of studies described intervention outcomes or reported quantitative data.

**Conclusion:**

This review highlights significant gaps in evidence-based interventions tailored to psychiatric care, as well as a lack of research from long-term care settings and low- and middle-income countries. To enhance patient safety in psychiatry, future efforts should prioritize the development and implementation of targeted strategies, multidisciplinary collaboration, integration with general patient safety initiatives, and robust quantitative evaluation. Strengthening safety culture across psychiatric facilities is essential to reduce harm and improve care quality for this high-risk population.

**Systematic Review Registration:**

https://www.crd.york.ac.uk/PROSPERO/, identifier CRD42023389235.

## 1 Introduction

Patient safety is a fundamental component of healthcare quality, aiming to prevent harm to patients during the provision of care. The year 2000 marked the publication of the seminal report “To Err is Human: Building a Safer Health System” (1) which transformed the landscape of patient safety research and practice by establishing standardized approaches to safety measurement and improvement in healthcare settings.

Despite the advancement of care, patient harm is still an extremely common event: it has been estimated that incidents occur in about 10% of hospitalizations, causing the loss of more than 75 DALY per 100000 inhabitants among OECD Countries (2, 3).

While extensive research has been conducted on patient safety in general hospital settings, less attention has been given to psychiatric inpatient facilities, where unique challenges and risks arise —including Patient Safety Incidents (PSIs) (i.e., events or circumstances that could have resulted, or did result, in unnecessary harm to a patient (4)).

Psychiatric inpatients often present with complex conditions that heighten their vulnerability to safety incidents (such as self-harm, aggression, medication errors, and falls) (5, 6), facing approximately double the risk of preventable adverse events compared to other patients (3). Additionally, factors such as communication barriers, restrictive interventions, and environmental hazards play a critical role in shaping patient safety outcomes within these units (5, 7). In psychiatric inpatient settings (i.e., specialized centers or departments where individuals with mental health conditions receive treatment and care on a residential basis), the occurrence of PSIs is of particular concern, due to the complex nature of mental health disorders (8).

Despite the growing interest toward patient safety in the last years, evidence from psychiatry remains limited. Indeed, while investigations into various aspects of quality of care are abundant, specifically related to the theme of safety (5), studies specifically addressing PSIs in mental health settings remain limited (9), with less than 25% of worldwide countries that have implemented patient safety initiatives in this context (3). In this regard, an exploratory systematic review conducted by Thibaut et al. (5) in 2019, synthesized evidence on patient safety in the inpatient psychiatric setting. Although this review provided an important overview of patient safety in the inpatient psychiatric setting, it is worth mentioning that it solely included studies in which patient safety was the main outcome, research question, or central aim, potentially overlooking studies where patient safety was analyzed within broader healthcare discussions rather than as a primary focus. Since the publication of that review over five years ago, additional studies have substantially expanded the body of literature on patient safety incidents in psychiatric inpatient care.

To address this gap in knowledge, we conducted a systematic review aimed at providing a more comprehensive and nuanced synthesis, integrating recent evidence to deepen the understanding of the typologies, determinants, and preventive strategies associated with PSIs within psychiatric inpatient settings. Specifically, our review sought to identify common types of PSIs reported in the literature, their associated determinants (i.e., contributing factors), preventive strategies aimed at reducing risk, consequences of these incidents on patients and healthcare systems, and coping mechanisms employed by institutions.

## 2 Materials and methods

### 2.1 Search strategy

We conducted a systematic review of the literature according to the Recommendations of the Preferred Reporting Items for Systematic Reviews and Meta-Analyses statement (10). The complete review protocol is available on PROSPERO (ID: CRD42023389235). The search was performed on PubMed and Scopus databases on 6 December 2024. The search strings were developed considering the following PICOS (Patients, Interventions, Comparators, Outcomes, and Study design) criteria: (P) Psychiatric adult (18 years or older) inpatients, (I) Patient safety incidents (namely, any unintended or unexpected incident which could have, or did, lead to harm for one or more patients receiving healthcare - NHS England), (C) no control group, (O) emerging determinant, strategies, and consequences on patient safety incidents in psychiatric inpatient population. We included studies published in English, Spanish, and Italian languages, conducted on original data (i.e., primary research), and published from 2000 onwards, as this year marked the publication of the report “To Err is Human: Building a Safer Health System” (1). Since our research aimed to focus on adults, we excluded studies conducted on children, adolescents, and subjects under the age of 18 years. In addition, we excluded studies on patients affected by Parkinson’s disease, Alzheimer’s disease, or dementia as they cannot be considered psychiatric-only disorders (11). Finally, we excluded all type of articles without primary data like reviews, as well as commentaries, study protocols, and opinion papers.

Search strings (**Table 1**) combine three blocks of terms: a first one with terms referring to PSIs, the second one for the context, and the third one containing keyword for excluding non-relevant records.

**Table 1:** Search strings. The search was performed on December 6^th^, 2024.

Database (retrieved records)	String
PubMed (1357)	(“adverse event” OR “critical incidents” OR “medical error” OR (“medication error”) OR “involuntary treatment” OR “near miss” OR “patient safety” OR “sentinel event” OR “second victim”) AND ((psychogeriatric OR psychiatric OR psychiatry OR forensic OR “mental health”) AND (hospital OR “inpatient” OR “inpatients” OR ward OR unit OR “clinic” OR service OR facilities OR facility OR nurses)) NOT (community OR neonatal OR young OR child OR children OR adolescent OR youth OR student OR “COVID-19” OR coronavirus OR ambulance OR home OR alzheimer OR dementia OR anaesthesiology OR parkinson OR surgery OR acupuncture OR endoscopy OR intravenous OR migraine OR “emergency department” OR depression OR rehabilitation OR dna OR rna OR cardiac OR cardiology OR liposome OR hypertension OR antimicrobial OR epilepsy OR epileptic OR spinal OR dialysis OR influenza OR COPD OR cancer OR diabetes OR diabetic) AND (“2000”[Date - Publication]: “3000”[Date - Publication])
Scopus (2928)	(“adverse event” OR “critical incidents” OR “medical error” OR (“medication error”) OR “involuntary treatment” OR “near miss” OR “patient safety” OR “sentinel event” OR “second victim”) AND ((psychogeriatric OR psychiatric OR psychiatry OR forensic OR “mental health”) AND (hospital OR “inpatient” OR “inpatients” OR ward OR unit OR “clinic” OR service OR facilities OR facility OR nurses)) AND NOT (community OR neonatal OR young OR child OR children OR adolescent OR youth OR student OR “COVID-19” OR coronavirus OR ambulance OR home OR alzheimer OR dementia OR anaesthesiology OR parkinson OR surgery OR acupuncture OR endoscopy OR intravenous OR migraine OR “emergency department” OR depression OR rehabilitation OR dna OR rna OR cardiac OR cardiology OR liposome OR hypertension OR antimicrobial OR epilepsy OR epileptic OR spinal OR dialysis OR influenza OR COPD OR cancer OR diabetes OR diabetic) AND PUBYEAR > 1999 AND (LIMIT-TO (SUBJAREA,”MEDI”) OR LIMIT-TO (SUBJAREA,”NURS”) OR LIMIT-TO (SUBJAREA,”PSYC”) OR LIMIT-TO (SUBJAREA,”HEAL”)) AND (LIMIT-TO (LANGUAGE,”English”)) AND (LIMIT-TO (LANGUAGE,”Spanish”) OR LIMIT-TO (LANGUAGE,”English”) OR LIMIT-TO (LANGUAGE,”Italian”))
Embase (1813)	(‘adverse event’/exp OR ‘adverse event’ OR ‘critical incidents’ OR ‘medical error’/exp OR ‘medical error’ OR ‘medication error’/exp OR ‘medication error’ OR ‘involuntary treatment’/exp OR ‘involuntary treatment’ OR ‘near miss’ OR ‘patient safety’/exp OR ‘patient safety’ OR ‘sentinel event’/exp OR ‘sentinel event’ OR ‘second victim’/exp OR ‘second victim’) AND (psychogeriatric OR psychiatric OR ‘psychiatry’/exp OR psychiatry OR forensic OR ‘mental health’/exp OR ‘mental health’) AND (‘hospital’/exp OR hospital OR ‘inpatient’/exp OR ‘inpatient’ OR ‘inpatients’/exp OR ‘inpatients’ OR ‘ward’/exp OR ward OR ‘unit’/exp OR unit OR ‘clinic’/exp OR ‘clinic’ OR service OR facilities OR facility OR ‘nurses’/exp OR nurses) NOT (‘community’/exp OR community OR neonatal OR young OR ‘child’/exp OR child OR ‘children’/exp OR children OR ‘adolescent’/exp OR adolescent OR ‘youth’/exp OR youth OR ‘student’/exp OR student OR ‘covid-19’/exp OR ‘covid-19’ OR ‘coronavirus’/exp OR coronavirus OR ‘ambulance’/exp OR ambulance OR ‘home’/exp OR home OR alzheimer OR ‘dementia’/exp OR dementia OR ‘anaesthesiology’/exp OR anaesthesiology OR parkinson OR ‘surgery’/exp OR surgery OR ‘acupuncture’/exp OR acupuncture OR ‘endoscopy’/exp OR endoscopy OR intravenous OR ‘migraine’/exp OR migraine OR ‘emergency department’/exp OR ‘emergency department’ OR ‘depression’/exp OR depression OR ‘rehabilitation’/exp OR rehabilitation OR ‘dna’/exp OR dna OR ‘rna’/exp OR rna OR ‘cardiac’/exp OR cardiac OR ‘cardiology’/exp OR cardiology OR ‘liposome’/exp OR liposome OR ‘hypertension’/exp OR hypertension OR ‘antimicrobial’/exp OR antimicrobial OR ‘epilepsy’/exp OR epilepsy OR ‘epileptic’/exp OR epileptic OR spinal OR ‘dialysis’/exp OR dialysis OR ‘influenza’/exp OR influenza OR ‘copd’/exp OR copd OR ‘cancer’/exp OR cancer OR ‘diabetes’/exp OR diabetes OR ‘diabetic’/exp OR diabetic) AND (2000:py OR 2001:py OR 2002:py OR 2003:py OR 2004:py OR 2005:py OR 2006:py OR 2007:py OR 2008:py OR 2009:py OR 2010:py OR 2011:py OR 2012:py OR 2013:py OR 2014:py OR 2015:py OR 2016:py OR 2017:py OR 2018:py OR 2019:py OR 2020:py OR 2021:py OR 2022:py OR 2023:py OR 2024:py OR 2025:py) AND [adult]/lim

### 2.2 Quality appraisal

The risk of bias in the included studies was assessed using the JBI framework’s established guidelines. Specifically, the risk of bias of the included studies was evaluated using JBI checklists for qualitative studies (12), case series (13), quasi-experimental study (14), narrative (15), prevalence (16), and cross-sectional studies (17), as well as the MMAT tool for mixed-methods studies (18). Given the potentially large number of included reports, the quality appraisal was conducted adopting an innovative artificial intelligence-driven approach. Specifically, we used the GPT-5 mini large language model (LLM), accessed via application programming interfaces provided by OpenRouter using a purposely developed R script. Full-texts were pre-processed as images in order to allow the LLM to access also tables and images. As manual accuracy validation, this approach was piloted on 15% of the included studies, which were evaluated both by the LLM and a human researcher (AM), while a second human researcher (AC) checked the inconsistencies. The detailed methodology (including the source code, prompts, and pilot results) are available in the **Supplementary Material**. Overall quality was established as follows: studies scored 70% or lower were classified as low quality, between 70 and 79% as medium quality, between 80 and 90% high quality, and greater than 90% excellent quality (19).

### 2.3 Data extraction and classification

Retrieved records were entered on a Google Sheet database; two researchers (AC and SR) independently screened the articles to assess studies’ eligibility for inclusion, and inconsistencies were resolved after a discussion that involved all the research team members. After eligibility screening, three researchers (AC, SR, MR) extracted the study characteristics from the full texts. In detail, the following information was extracted: study design and setting, country, population, sample size, study aim, principal topic, identified PSIs and related determinants, preventive strategies, consequences, and coping strategies. As a common data extraction line, only the information explicitly reported, mentioned or discussed in the included studies was extracted, without additional judgement or inference from the researchers. Given the conceptual focus of our synthesis, the absence of quantitative outcome pooling, and the emerging and scarce nature of the literature on this topic, we opted to include all available evidence based on thematic relevance, while a formal risk-of-bias assessment would have offered limited interpretive utility.

### 2.4 Data classification and visualization

Since, as known by the Authors, no international and comprehensive classification of patient safety has been specifically developed for the psychiatric context, we adopted the Conceptual Framework for the International Classification for Patient Safety (ICPS) (20) to classify and therefore present emerging information. This framework, developed by the WHO in 2009, has been specifically designed to provide a comprehensive understanding of the patient safety domain through the assessment of the different aspects (e.g., risk identification, prevention, detection, and reduction) (3).

To perform information classification, we built a relational database using the AirTable software. Specifically, the table containing the previously extracted studies characteristics was imported and a unique identifier (DOI, if available; otherwise, a progressive identification number) was attributed to each record. Therefore, a second table (i.e., “relation table”) was built to map and classify the information on PSI. This second table contained the following columns: “study identifier”, “incident type category”, “incident type subcategory”, “contributing factor category”, contributing factor subcategory”, “actions to reduce risk category”, “actions to reduce risk subcategory”, “mitigating factors category”, and “mitigation factors subcategory”. In this relation table, each identified relation was entered as a single database line. For example, if a study identified two different determinants for “falls”, this results in two entries referring to the same study identifier. The abovementioned categories and subcategories refer to the ICPS taxonomy. Indeed, each PSI identified in the included studies was classified according to the Incident Type taxonomy, which classifies “incidents of a common nature grouped because of shared, agreed features” (20). The same approach was used to classify determinants as Contributing Factors, namely “the reason, situational factor, or latent condition that played a role in the genesis of an adverse outcome”, preventive strategies as Actions to Reduce Risk (meaning structured proactive strategies or programs aimed at preventing PSIs) and coping strategies in Mitigating Factors (“An action or circumstance which moderates the progression of an incident towards harming a patient”). This classification was performed by a researcher (AC) and therefore checked by a second one (SR). Inconsistencies were solved through a discussion involving the entire research team. Additionally, we collected quantitative information related to the occurrence rate of diverse PSIs in a third table on the database. Specifically, we expressed the incidence as the number of events per 1000 bed-day. This information was gathered directly from the full text or, when feasible with available information, calculated by the researchers. Moreover, we collected information about the proactive interventions described in the included studies.

To facilitate the interpretation of classification results, Sankey flow diagrams were used to visualize the identified relations. Sankey diagrams are a data visualization approach particularly useful to highlight the relation between different categories or conditions (21). A Sankey diagram includes several *nodes* representing states, which are grouped in *stages* on the basis of one or more characteristics. The *size* of each node is determined by the magnitude (e.g., number of objects) of the state. Nodes are interconnected, according to their relations, with *arcs*. In our study we considered as nodes each classified PSIs, determinants, consequences, preventive and mitigation strategies. Their size represents the number of times they are mentioned, and the arcs the relations identified in the included studies. Finally, stages are represented by the WHO International Classification classes (i.e., Actions to reduce risk, Contributing Factors, Incident Types, and Mitigating Factors). Sankey diagrams were plotted using R 4.5.2 with the *ggsankey* library.

## 3 Results

**Figure 1:**
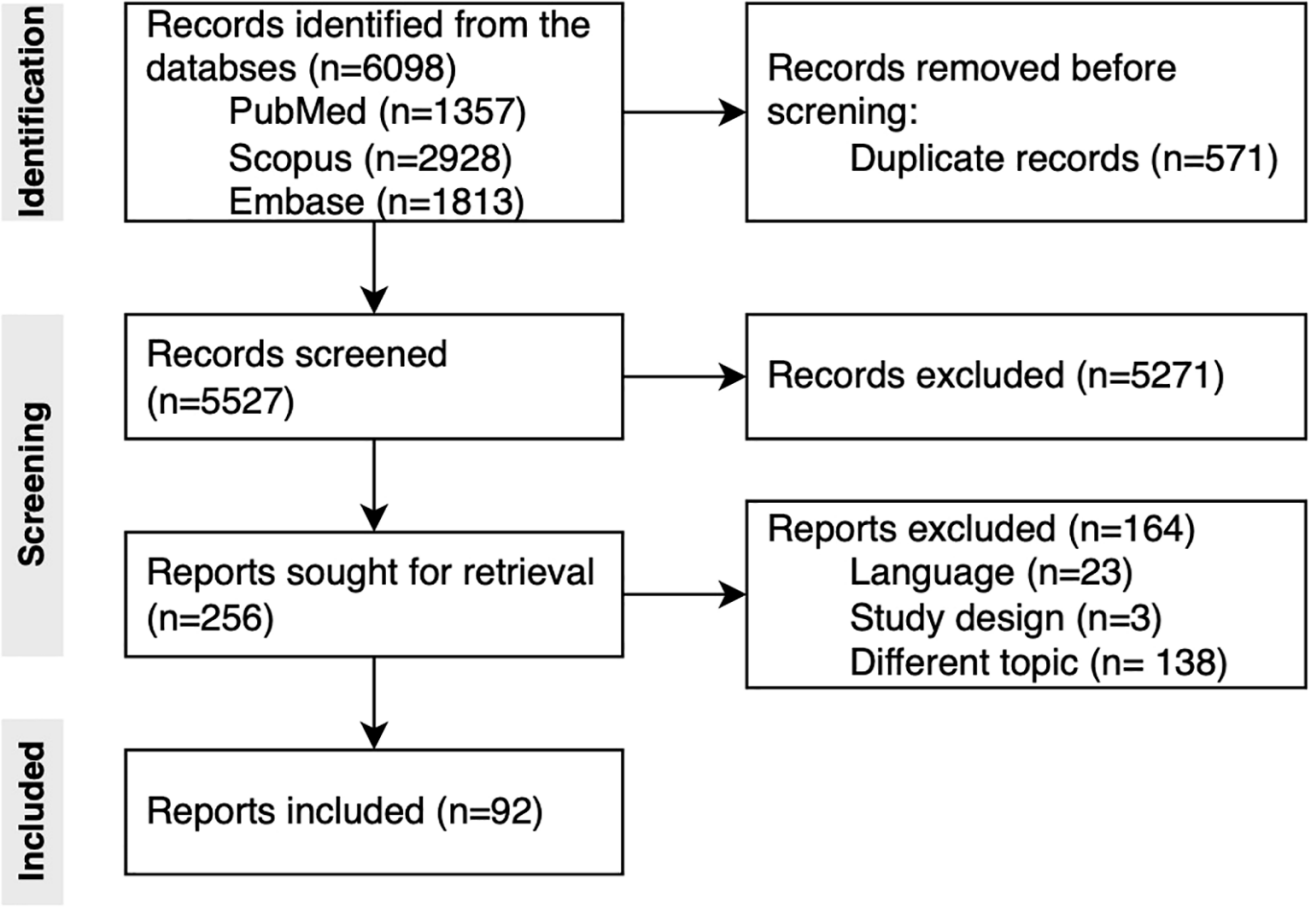
PRISMA flowchart.

After duplicates removal, 5527 titles and abstracts were screened. Of them, 5271 were excluded. A total of 256 records underwent full-text screening, and 163 reports were excluded for dealing with a different topic (n=137), and for not matching language (n=23) and study design (n=3) inclusion criteria. The PRISMA flowchart is shown in **Figure 1**, while the details of the included studies are shown in **Table 2**. Complete PRISMA checklist are available in **Supplementary Material**. Of the 92 included studies, 29 were descriptive (i.e., 20 prevalence studies, 5 case series, 3 narrative evidence, 1 case report) 23 qualitative, 13 cross-sectional, 13 quasi-experimental, 8 mixed-methods, 5 observational (4 cohort and 1 case-control studies), and 1 Delphi study. Studies were mainly conducted in the United States (n= 29), United Kingdom (n=14), Sweden (n=6), and Australia (n=5). Only two studies (22, 23) were conducted in the African region, while no records were retrieved from South America. Three studies were conducted worldwide (24–26). Study settings were hospitals (n=62), other non-specified mental health institutions (n=14), veteran affairs institutions (n=9), long term care (n=4), and forensic units (n=3). The studied populations were mainly healthcare workers (e.g., doctors, nurses, psychiatrists) (n=41) and psychiatric patients (n=36), while reports (e.g., incident reports and root cause analyses) and facilities were the subjects of 10 and 6 studies, respectively. When it comes to quality, the majority of the studies were classified as excellent or high quality (29% and 25%, respectively), while 32% of the studies were graded as medium quality and 14% as low quality.

**Table 2:** Characteristics of the included studies.

Study	Country	Study design	Setting	Main topic	Population (type, sample size)	Overall quality
Abela-Dimech et al., 2017 (69)	Canada	Quasi-experimental	Hospital	Patient safety in psychiatry	N/A (N/A)	Low
Abraham et al., 2016 (84)	United States	Descriptive cross-sectional	Hospital	Patient safety in psychiatry	W (120)	Low
Adler et al., 2020 (68)	United States	Qualitative	Hospital	Patient safety in psychiatry	P (28)	Excellent
Alenzi et al., 2024 (128)	Saudi Arabia	Descriptive cross-sectional	Hospital	Patient safety in psychiatry	R (23355)	Excellent
Alshehri et al., 2021 (129)	United Kingdom	Mixed-methods	Hospital	Patient safety in psychiatry	R (94571)	Excellent
Anderson et al., 2012 (61)	United Kingdom	Qualitative	Hospital	Patient safety in general	W (31)	High
Asikainen et al., 2020 (71)	Finland	Qualitative	Hospital	Patient safety in psychiatry	W (54)	High
Bayramzadeh et al., 2017 (28)	United States	Mixed-methods	Hospital	Patient safety in psychiatry	F (9)	Excellent
Bayramzadeh et al., 2019 (29)	United States	Case series	Hospital	Setting safety in psychiatry	P (N/A)	High
Beaglehole et al., 2017 (34)	New Zealand	Quasi-experimental	Hospital	Setting safety in psychiatry	P (64)	Medium
Berzins et al., 2020 (70)	United Kingdom	Qualitative	Mental health (not specified)	Patient safety in psychiatry	P, W (20)	Low
Biswas et al., 2023 (58)	United States	Descriptive cross-sectional	Forensic Unit	Patient safety in psychiatry	P (220)	Excellent
Boussat et al., 2015 (27)	France	Descriptive cross-sectional	Hospital	Patient safety in psychiatry	W (22)	Low
Brady et al., 2012 (30)	United States	Quasi-experimental	Hospital	Patient safety in psychiatry	W (16)	Medium
Brooker et al., 2024 (130)	United Kingdom	Descriptive cross-sectional	Mental health (not specified)	Patient safety in psychiatry	F (38)	Low
Cardone et al., 2009 (56)	Italy	Descriptive	Mental health (not specified)	Setting safety in general	P (20)	Excellent
Chen et al., 2012 (47)	Taiwan	Cohort study	Hospital	Patient safety in psychiatry	P (75)	Low
Chen et al., 2024 (131)	China	Cross-sectional	Hospital	Impact of PSI on psychiatric staff	W (9744)	Excellent
Cook et al., 2020 (40)	United Kingdom	Cross-sectional	Hospital	Impact of PSI on psychiatric staff	P, W (N/A)	Low
Costopoulos et al., 2019 (132)	United States	Cohort study	Hospital		P (4936)	High
Derblom et al., 2021 (133)	Sweden	Qualitative	Hospital	Patient safety in psychiatry	W (10)	High
Deringer et al., 2014 (43)	United States	Mixed-methods	Mental health (not specified)	Impact of PSI on psychiatric staff	W (21)	Low
Digby et al., 2020 (72)	Australia	Qualitative	Hospital	Impact of PSI on psychiatric staff	W (24)	High
Dückers et al., 2021 (24)	Worldwide	Cross-sectional	Mental health (not specified)	Patient safety in psychiatry	N/A (N/A)	Excellent
Duxbury et al., 2010 (75)	United Kingdom	Qualitative	Hospital	Setting safety in general	P, W (N/A)	High
Glantz et al., 2019 (134)	Sweden	Descriptive cross-sectional	Mental health (not specified)	Setting safety in psychiatry	W (12)	Low
Grasso et al., 2001 (77)	United States	Descriptive	Hospital	Patient safety in psychiatry	N/A (N/A)	Excellent
Harrington et al., 2019 (35)	Australia	Quasi-experimental	Hospital	Patient safety in psychiatry	P (263)	Low
Instefjord et al., 2014 (50)	Norway	Cross-sectional	Hospital	Patient safety in psychiatry	W (34)	Low
Ito et al., 2003 (32)	Japan	Descriptive cross-sectional	Long-term care	Patient safety in psychiatry	F (221)	Low
Jayaram et al., 2011 (33)	United States	Qualitative	Hospital	Patient safety in psychiatry	N/A (N/A)	High
Kallman et al., 2022 (45)	Sweden	Cross-sectional	Hospital	Patient safety in general	F (1476)	High
Keers et al., 2014 (76)	United Kingdom	Descriptive cross-sectional	Hospital	Patient safety in psychiatry	P (N/A)	High
Keers et al., 2015 (51)	United Kingdom	Cross-sectional	Hospital	Patient safety in psychiatry	N/A (274)	Excellent
Kelly et al., 2011 (80)	Australia	Qualitative	Mental health (not specified)	Patient safety in psychiatry	P, W (22)	High
Koukia et al., 2009 (135)	Greece	Qualitative	Hospital	Patient safety in psychiatry	W (103)	Medium
Kracher et al., 2020 (73)	United States	Quasi-experimental	Hospital	Patient safety in general	P (N/A)	Low
Krvavac et al., 2023 (60)	Norway	Cohort study	Hospital	Patient safety in psychiatry	P (278)	High
Kuosmanen et al., 2019 (44)	Finland	Quasi-experimental	Forensic Unit	Patient safety in psychiatry	P, W (283)	Excellent
Kuosmanen et al., 2021 (36)	Finland	Qualitative	Hospital	Patient safety in psychiatry	F (2521)	Medium
Kuusisto et al., 2021 (79)	Finland	Cross-sectional	Hospital	Patient safety in general	N/A (N/A)	Medium
Kwobah et al., 2023 (23)	Kenia	Qualitative	Hospital	Patient safety in psychiatry	W (26)	Medium
Lebas et al., 2024 (136)	France	Descriptive cross-sectional	Hospital	Patient safety in psychiatry	R (609)	Low
Lees et al., 2012 (83)	United States	Case series	Hospital	Patient safety in psychiatry	N/A (76)	Medium
Lessard‐Deschênes et al., 2021 (86)	Canada	Qualitative	Forensic Unit	Patient safety in psychiatry	P (15)	High
Lindwall et al., 2011 (137)	Sweden	Qualitative	Mental health (not specified)	Patient safety in psychiatry	W (16)	Excellent
Lykkegaard Soerensen et al., 2013 (138)	Denmark	Descriptive cross-sectional	Hospital	Patient safety in psychiatry	P (67)	Low
Maddineshat et al., 2024 (46)	Iran	Qualitative	Mental health (not specified)	Patient safety in psychiatry	W (23)	Medium
Marcus et al., 2018 (139)	United States	Descriptive cross-sectional	Veteran Affairs	Patient safety in psychiatry	P (8052)	Excellent
Marcus et al., 2021 (87)	United States	Descriptive	Mental health (not specified)	Patient safety in psychiatry	W (N/A)	Excellent
Mascherek et al., 2016 (53)	Switzerland	Delphi	Mental health (not specified)	Patient safety in psychiatry	W (13)	Excellent
Mills et al., 2010 (54)	United States	Quasi-experimental	Veteran Affairs	Patient safety in psychiatry	F (N/A)	Excellent
Mills et al., 2013 (140)	United States	Descriptive cross-sectional	Veteran Affairs	Setting safety in psychiatry	R (471)	High
Mills et al., 2018 (55)	United States	Case series	Veteran Affairs	Patient safety in psychiatry	R (87)	Medium
Mills et al., 2020 (62)	United States	Quasi-experimental	Hospital	Patient safety in psychiatry	P (389)	Low
Mitchell et al., 2001 (52)	United Kingdom	Qualitative	Mental health (not specified)	Impact of PSI on psychiatric staff	W (23)	Excellent
Ndebele et al., 2024 (81)	United Kingdom	Mixed-methods	Hospital	Patient safety in psychiatry	P, W (39)	Low
Norris et al., 2021 (63)	United States	Descriptive cross-sectional	Veteran Affairs	Patient safety in general	P (82)	Low
Okkenhaug et al., 2019 (41)	Norway	Cross-sectional	Hospital	Patient safety in psychiatry	R (240)	Medium
Olashore et al., 2018 (22)	Botswana	Descriptive cross-sectional	Hospital	Impact of PSI on psychiatric staff	W (201)	Excellent
Olasoji et al., 2024 (141)	Australia	Qualitative	Hospital	Patient safety in psychiatry	W (8)	Medium
Park et al., 2023 (48)	South Korea	Cross-sectional	Hospital	Patient safety in psychiatry	W (157)	Excellent
Pelto-Piri et al., 2020 (64)	Sweden	Mixed-methods	Hospital	Impact of PSI on psychiatric staff	R (283)	Excellent
Powell-cope et al., 2014 (85)	United States	Qualitative	Hospital	Patient safety in psychiatry	W (25)	High
Price et al., 2024 (65)	United Kingdom	Qualitative	Hospital	Patient safety in psychiatry	P, W (46)	Excellent
Quigley et al., 2014 (31)	United States	Quasi-experimental	Hospital	Patient safety in psychiatry	W (77)	Low
Raboch et al., 2010 (142)	Greece	Cross-sectional	Hospital	Patient safety in psychiatry	W (2030)	Low
Raveendranathan et al., 2012 (143)	India	Case series	Hospital	Patient safety in psychiatry	P (100)	Excellent
Reilly et al., 2019 (91)	United States	Descriptive cross-sectional	Veteran Affairs	Impact of PSI on psychiatric staff	R (583)	Excellent
Renwick et al., 2019 (144)	United Kingdom	Cross-sectional	Hospital	Impact of PSI on psychiatric staff	W (384)	High
Riblet et al., 2019 (145)	United States	Cohort study	Veteran Affairs	Patient safety in psychiatry	R (39)	Low
Roos af Hjelmsater et al., 2019 (57)	Sweden	Case series	Mental health (not specified)	Patient safety in general	P (436)	High
Rotschild al., 2007 (39)	United States	Descriptive cross-sectional	Hospital	Patient safety in psychiatry	P (1559)	High
Russ et al., 2017 (146)	United States	Case report	Hospital	Impact of PSI on psychiatric staff	P (1)	Medium
Russotto et al., 2024 (26)	Worldwide	Descriptive cross-sectional	Hospital	Patient safety in psychiatry	W (33)	Excellent
Sawamura et al., 2005 (147)	Japan	Cross-sectional	Long-term care	Patient safety in psychiatry	R (221)	Medium
Schwappach et al., 2019 (148)	Switzerland	Descriptive cross-sectional	Hospital	Patient safety in psychiatry	W (817)	Low
Seeherunwong et al., 2022 (82)	Thailand	Case-control	Hospital	Patient safety in psychiatry	P (240)	Excellent
Shah et al., 2022 (67)	United Kingdom	Quasi-experimental	Hospital	Patient safety in psychiatry	P (38)	Low
Skelton et al., 2019 (78)	United Kingdom	Mixed-methods	Hospital	Patient safety in psychiatry	W (24)	High
True et al., 2017 (42)	United States	Qualitative	Hospital	Patient safety in psychiatry	W (20)	High
Vahidi et al., 2018 (59)	Iran	Qualitative	Hospital	Patient safety in psychiatry	P, W (26)	High
Vargas et a et al., 2023 (149)	United States	Descriptive cross-sectional	Hospital	Patient safety in psychiatry	P (72)	Low
Varpula et al., 2023 (25)	Worldwide	Mixed-methods	Mental health (not specified)	Patient safety in psychiatry	N/A (N/A)	Excellent
Vermeulen et al., 2019 (150)	The Netherlands	Qualitative	Hospital	Patient safety in psychiatry	P, W (28)	High
Vruwink et al., 2022 (37)	The Netherlands	Mixed-methods	Long-term care	Patient safety in psychiatry	P (184)	Excellent
Watts et al., 2012 (66)	United States	Quasi-experimental	Veteran Affairs	Patient safety in psychiatry	P (25)	Low
Watts et al., 2017 (74)	United States	Quasi-experimental	Veteran Affairs	Patient safety in psychiatry	P (29)	Low
Whitecross et al., 2020 (38)	Australia	Quasi-experimental	Long-term care	Patient safety in psychiatry	P (981)	Low
Wurst et al., 2013 (151)	Germany	Cross-sectional	Hospital	Impact of PSI on psychiatric staff	W (226)	Low
Xu et al., 2022 (152)	China	Descriptive cross-sectional	Hospital	Impact of PSI on psychiatric staff	W (683)	Low
Zola et al., 2024 (153)	South Africa	Qualitative	Hospital	Impact of PSI on psychiatric staff	W (12)	High

(F, facility; P, psychiatric patients; R, incident reports; W, healthcare workers).

Only for 13 studies (27–39) (14%) it was possible to retrieve the incidence of diverse PSIs, which ranged from 0.2 (medication prescription incidents) to 8.4 (verbal aggressions by patient) events/1000 bed-days. Mean incident rates are reported and compared with those from general medical settings in **Table 3**.

**Table 3:** PSI incidence, expressed as number of events per 1000 patient-days (mean of values obtained from included studies).

PSI	Number of studies	Mean incidence (events/1000 patient-days) in inpatient psychiatry	Incidence (events/1000 patient-days) in general medical settings
Verbal aggression by patient	2 (36, 38)	8.4	N/A
Physical aggression by patient	7 (28, 30, 34–38)	7.3	N/A
Falls	5 (27–31)	6.8	6.1 (154)
Patient inappropriate behavior	5 (27, 28, 34, 35, 38)	3.6	N/A
Medication incidents	5 (27, 30, 32, 33, 39)	4.0	9.7 (155)
Administration	7 (32, 33)	0.5	
Monitoring	1 (33)	0.3	
Preparation	2 (32, 33)	0.6	
Prescription	2 (32, 33)	0.2	
Transcription	1 (33)	3.1	
Sexual aggression by patient	2 (35, 38)	1.0	N/A
Self-harm/Suicide	1 (36)	0.8	< 0.1 (156)
Others	2 (27, 28)	0.1	N/A

N/A: it was not possible to retrieve this information from general medical setting.

**Figure 2:**
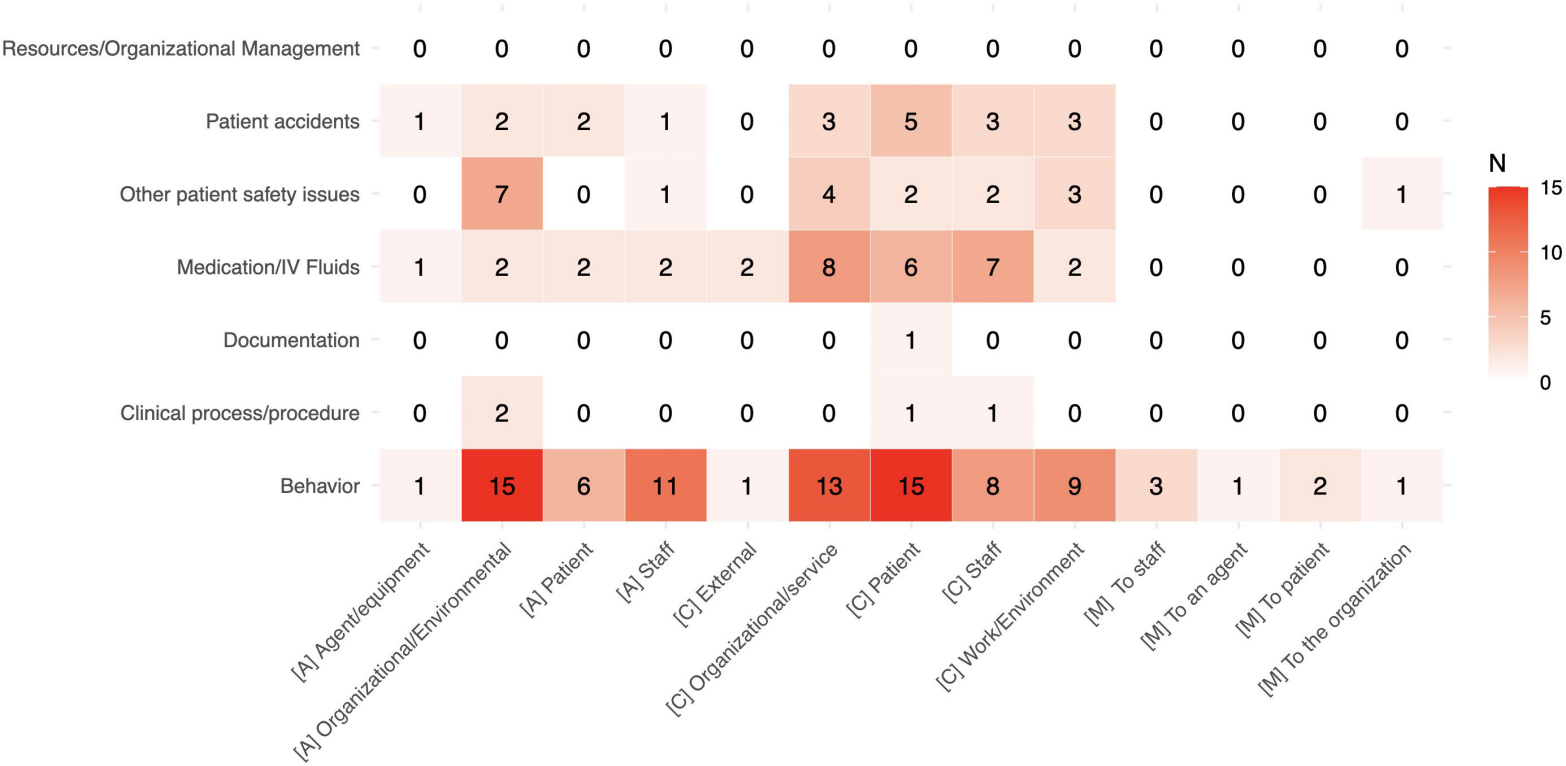
Heatmap showing the number of studies (N) mentioning the identified contributing factors ([C]), actions to reduce risk ([A]), and mitigating factors ([M]) for each ITC.

**Figure 3:**
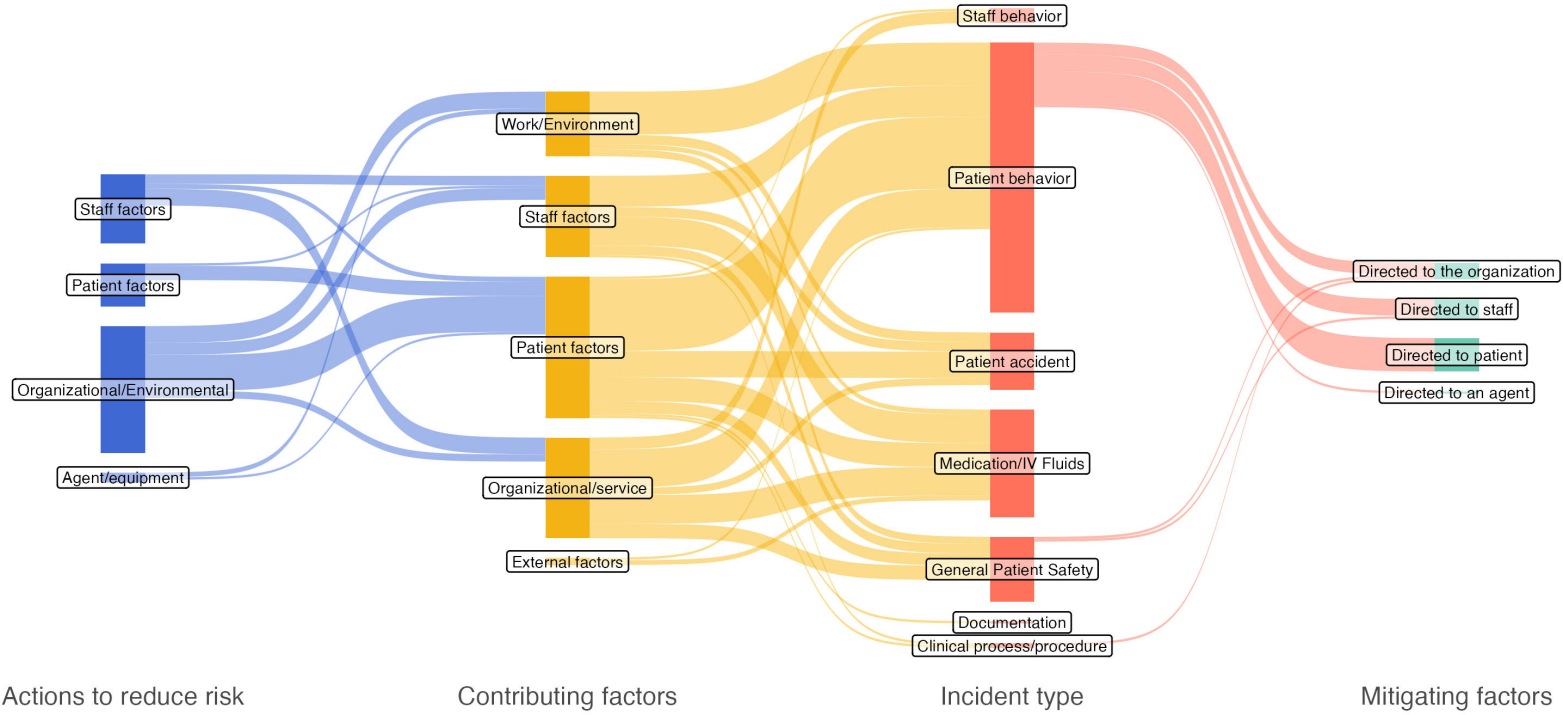
Sankey diagram of the identified Actions to reduce risk, Contributing factors, Incident types, and Mitigating factors. The size of each element is proportional to the number of studies which mention it.

Complete data extraction and classification is shown in **Table 4**, while **Figure 2** summarizes the identified contributing factors, actions to reduce risk, and mitigating factors for each ITC. 81 (88%) studies described one or more PSI, dealing with six out of the 13 ICPS ITCs. More specifically, the represented categories were “Behavior” (n=56), “Medication/IV fluids” (n=20), “Patient accidents” (n=15)”, “Clinical Process/Procedure” (n=4), “Documentation” (n=2), and “Resources/Organizational Management” (n=2). No evidence was found regarding PSI from the “Clinical administration”, “Healthcare associated infections”, “Blood/blood products”, “Nutrition”, “Oxygen/gas/vapor”, “Medical device/equipment”, and “Infrastructure/building/fixtures” ITCs. Contributing factors and actions to reduce risk were identified in 53 (58%) and 37 (40%) studies, respectively, while mitigating factors were described only in eight (9%) studies. Although 11 studies (26, 40–49) addressed patient safety in psychiatric inpatient settings, they did not clearly specify PSI. Consequently, we created an “General Patient Safety” category to classify these studies. Detailed description of contributing factors, actions to reduce risk, mitigating factors, and interventions for diverse ITC (i.e., behavior-related, medication, patient accidents, and clinical processes and procedures incidents) is available in the following sections, while the complete Sankey flow diagram is shown in **Figure 3**. Evidence on some PSIs was limited. Specifically, incidents related to documentation and those concerning resources and organizational management were each reported in two studies (50–53) and these studies often lacked detailed analysis of contributing factors, preventive strategies, or patient and organizational outcomes.

**Table 4:** WHO ICPS-based classification of emerging evidence from the included studies.

ITC	Patient safety incident	Contributing factors	Actions to reduce risk	Mitigating factors
Behavior (n=57)	Patient intended self-harm/suicide (n=28)	**External factors:** Services, systems, and policies (n=1) **Organizational/service factors:** Organizational decision/culture (n=1) Protocols/policies/procedures/processes (n=5) Resources workload (n=2) **Patient factors:** Behavior (n=2) Social (n=4) Disease (n=2) **Staff factors:** Performance (n=3) Behavior (n=4) Social (n=1) **Work/environment factors:** Physical environment/infrastructure (n=6)	**Organizational/environmental factors:** Arranging ready access to protocol (n=1) Performing risk assessment (n=2) Matching physical environment to needs (n=2) **Patient factors:** Provision of adequate care/support (n=1) Provision of monitoring equipment (n=1) **Staff factors:** Adequate staff number (n=1) Availability of checklist (n=1) Improving safety culture (n=2) Training (n=44) **Agent/equipment factors:** Provision of equipment (n=1)	**Directed to the organization:** Effective protocol available (n=1) **Directed to an agent:** security/physical environment measure (n=1) **Directed to staff:** Relevant persons educated (n=1)
	Physical assault by patient (n=13)	**Organizational/service factors:** organizational decision/culture (n=1) Organization of teams (n=1) **Patient factors:** Behavior (n=2) Disease (n=2) Social (n=2) **Staff factors:** Social (n=1) Performance (n=1) **Work/environment factors:** Physical environment/infrastructure (n=3)	**Organizational/environmental factors:** improving safety culture (n=1) Matching physical environment to needs (n=1) **Patient factors:** Provision of patient education/training (n=1) Provision of adequate care/support (n=1) **Staff factors:** Adequate staff numbers/(n=2) Training (n=2)	**Directed to staff:** Relevant persons educated (n=1)
	Noncompliant/uncooperative/obstructive patient behavior (n=8)	**Patient factors:** Behavior (n=2) Social (n=1)	**Organizational/environmental factors:** Matching physical environment to needs (n=1) Improving safety culture (n=4) **Patient factors:** Provision of adequate care/support (n=1) Provision of patient education/training (n=1) Provision of protocols/decision support (n=1) **Staff factors:** Training (n=1)	**Directed to organization:** effective protocol available (n=1)
	Patient inconsiderate/rude/hostile/inappropriate behavior (n=7)	**Patient factors:** Cognitive (n=1) **Staff factors:** Cognitive (n=1) **Work/environment factors:** Physical environment/infrastructure (n=1)	**Organizational/environmental factors:** Matching physical environment to needs (n=1) Improving safety culture (n=1) **Patient factors:** Provision of patient education/training (n=1)	
	Sexual assault by patient (n=5)	**Work/environment factors:** Physical environment/infrastructure (n=1)	**Organizational/environmental factors:** Improving safety culture (n=1)	
	Patient wandering/absconding (n=2)	**Patient factors:** Behavior (n=1)	**Organizational/environmental factors:** Improving safety culture (n=2)	
	Verbal aggression by patient (n=2)	**Patient factors:** Behavior (n=1) Organizational/service-related: resources/workload (n=1)	**Staff factors:** Adequate staff number (n=1)	
	Risky/reckless/dangerous patient behavior (n=1)	**Patient factors:** Behavior (n=1)	**Organizational/environmental factors:** Improving safety culture (n=1)	**Directed to organization:** Effective protocol available (n=1)
	Harassment by patient (n=2)	**Staff factors:** Behavior (n=1) **Patient factors:** Social (n=1) Disease (n=1)		**Directed to staff:** Relevant person educated (n=1)
	Patient aggression toward an inmate object (n=1)	**Patient factors:** Behavior (n=1)	**Organizational/environmental factors:** Improving safety culture (n=1)	
	Staff sexual assault (n=1)			
	Staff noncompliant/uncooperative/obstructive behavior (n=1)	**Organizational/service factors:** resources/workload (n=1) **Patient factors:** Behavior (n=1)		
	Staff inconsiderate, rude, hostile or inappropriate behavior (n=1)			
	Patient behavior (not specified) (n=16)	**Organizational/service factors:** protocols/policies/procedures/processes (n=1) Resources/workload (n=1) **Patient factors:** Disease (n=2) Performance (n=1) **Staff factors:** Behavior (n=1) **Work/environment factors:** Physical environment/infrastructure (n=2)	**Organizational/environmental factors:** Adequate staff numbers/(n=2) Arranging ready access to protocols, policies, and decision support (n=2) Matching physical environment to needs (n=2) **Patient factors:** Provision of adequate care/support (n=1) **Agent/equipment factors:** Provision of equipment (n=1) Provision of patient education/training (n=1) **Staff factors:** Availability of checklists/protocols/policies (n=1) Training (n=2)	**Directed to patient:** Apology (n=1) Help called for (n=1) Management/treatment (n=1) Patient education/explanation (n=1) **Directed to staff:** Relevant persons educated (n=1)
	Staff behavior (not specified) (n=2)	**Organizational/service factors:** resources/workload (n=2) Organization of teams (n=2)	**Staff factors:** Adequate staff number (n=1)	
Medication/IV fluids (n= 20)	Prescription error (n=6)	**Staff factors:** Social (n=1) Performance (n=1)	**Agent/equipment:** Provision of equipment (n=1)	
	Adverse drug reaction after administration (n=2)	**Organizational/service factors:** Protocols/policies/procedures/processes (n=1) **Patient factors:** Disease (n=1) **Staff factors:** Cognitive (n=1) Communication (n=1) **Work/environment factors:** Physical environment/infrastructure (n=1)	**Patient factors:** Provision of adequate care/support (n=1) Provision of patient education (n=1) **Staff factors:** Availability of checklist, protocols, and policies (n=1) Provision of medication dispensing aid (n=1) Training (n=1)	
	Monitoring, omitted medicine or dose (n=3)	**Organizational/service factors:** Protocols/policies/procedures/processes (n=2)		
	Administration, wrong patient (n=2)		**Patient factors:** Provision of adequate care/support (n=1) Provision of monitoring equipment (n=1)	
	Administration, wrong dose/strength of frequency (n=3)	**Organizational/service factors:** Protocols/policies/procedures/processes (n=1)		
	Preparation/dispensing (n=1)			
	Administration, wrong route (n=1)			
	Administration (not specified) (n=8)	**External factors:** Products, technology and infrastructure (n=1) **Organizational/service factors:** Protocols/policies/procedures/processes (n=3) Resources/workload (n=1) **Patient factors:** Communication (n=1) **Staff factors:** Behavior (n=1) Cognitive (n=1) Communication (n=1)		
	Not specified (n=10)	**External factors:** Products, technology and infrastructure (n=1) **Patient factors:** Communication (n=1) Disease (n=2) **Staff factors:** Behavior (n=1) Cognitive (n=1) Communication (n=1) Performance (n=1) **Organizational/service factors:** Resources/workload (n=3) **Work/environment factors:** Physical environment/infrastructure (n=1)	**Organizational/environmental factors:** Arranging ready access to protocols, policies, and decision support (n=1) Improving safety culture (n=1) **Staff factors:** Supervision/assistance (n=1) Availability of checklists/protocols/policies (n=1)	
Patient accidents (n= 15)	Falls (n=15)	**Organizational/service factors:** Organization of teams (n=1) Resources/workload (n=1) **Patient factors:** Behavior (n=2) Performance (n=2) Disease (n=3) **Staff factors:** Performance (n=1) Communication (n=2) **Work/environment factors:** Physical environment/infrastructure (n=3)	**Agent/equipment factors:** provision of equipment (n=1) **Organizational/environmental factors:** Improving safety culture (n=1) Matching physical environment to needs (n=2) **Patient factors:** Provision of adequate care/support (n=2) **Staff factors:** Training (n=1)	
	Not specified (n=1)	**Organizational/service factors:** resources/workload (n=1) Staff factors: performance (n=1)		
Clinical process/procedure (n=4)	Incomplete/inadequate diagnosis (n=1)			
	Incomplete/inadequate treatment (n=2)			
	Incomplete/inadequate general care/management (n=1)			
	Treatment, not performed when indicated (n=1)	**Staff factors:** Behavior (n=1)	**Organizational/environmental factors:** Improving safety culture (n=1)	
	Not specified (n=1)	**Patient factors:** Behavior (n=1)	**Organizational/environmental factors:** Improving safety culture (n=1)	
Documentation (n=2)	Medical record, unclear information (n=1)	**Patient factors:** Disease (n=1)		
	Charts/medical records assessment/consultations, document missing or unavailable (n=1)			
Resources/organizational management (n=2)	Protocol/policy/procedure/guideline availability/adequacy (n=2)			
Other patient safety issues (n=15)	(not specified) (n=14)	**Patient factors:** Social (n=2) Disease (n=2) Behavior (n=1) **Staff factors:** Cognitive (n=1) Performance (n=2) Behavior (n=1) **Organizational/service factors:** protocols/policies/procedures/processes (n=3) Organization of teams (n=1) Resources/workload (n=2) **Work/environment factors:** Physical environment (n=3)	**Organizational/environmental factors:** Arranging ready access to protocols/policies/decision support (n=1) Improving safety culture (n=6) Improved leadership/guidance (n=1) **Staff factors:** Orientation (n=1)	**Directed to organization:** Effective protocol available (n=1) **Directed to staff:** Effective communication (n=1)

### 3.1 Behavior-related incidents

As aforementioned, the most described PSI were the behavior-related ones. These incidents were also the ones with more information about contributing factors, actions to reduce risk, and mitigating factors. Specifically, 28 studies dealt with intended self-harm and suicide of patients. Of them, 11 included the incidence. For such events, the most described contributing factors were related to the physical environment and to protocols, processes, and procedures. Indeed, several environmental aspects have been identified as potential risk factors for such events, for example the presence of hanging anchoring points (54) and materials and objects that can be used as ligatures or weapons (28, 54, 55). When it comes to organizational aspects, inadequate or non-continuous patient observation, the absence of specific protocols and procedures, or the lack of a detailed suicidal risk medical evaluation could contribute to PSI occurrence (47, 56, 57). In addition, also patient characteristics such as young age, higher education, and psychiatric symptoms (e.g., paranoidal behavior, personality disorders, and the presence of hallucinations) have been identified (47, 58). Finally, staff-related factors such as insufficient training, inadequate communication, and scarce workforce can act as contributing factors (47, 57). Staff training on suicide risk prevention and de-escalation techniques were the most identified action to reduce risk, as well as the development of specific protocols (47, 56, 59, 60). Only three studies identified mitigating factors, which include the availability of specific dedicated protocols (61), staff training (58), and environmental safety measures (62).

The second most described behavior-related PSI was the physical assault by a patient (13 studies). Contributing factors were similar to those one identified for suicide/self-harm incidents, namely environmental-related (e.g., hanging anchoring points, objects that can be used as ligatures) (29, 36, 63), organizational-related (e.g., inadequate protocols and intermittent observation) (64), and staff-related (e.g., inadequate training, insufficient workforce and inability to apply de-escalation techniques) (64). When it comes to actions to reduce risk, training, adequate staff number, and the improvement of safety culture through dedicated protocols were pointed out as potential strategies (28, 64, 65). Notably, staff training was also the only reported mitigating factor (58), indicating that training was perceived as beneficial in both preventing incidents and in reducing their impact when they occurred. However, only one study pointed out potential mitigating factors (i.e., staff training) (58).

The third most described PSI was the patient’s noncompliant, uncooperative, obstructive behavior (eight studies). In contrast, the two abovementioned PSI, evidence on factors and actions was limited. Patient characteristics, such as long stay, alcohol and substance abuse, and history of seclusions (37, 61) were identified as potential contributing factors (37, 61, 66). Also, an inadequate physical environment which does not allow monitoring has been identified as a potential risk factor (28). Actions to reduce risk were mainly focused on providing adequate patient training, care, and decision support (67, 68). In contrast, the only mitigating factor described was the availability of specifically developed protocols and procedures (61). Analogue contributing factors and action to reduce risk were identified for patient inconsiderate or hostile behavior (28, 69). It is worth mentioning that two qualitative studies (23, 70) described PSIs caused by staff towards patients, specifically verbal, physical and sexual aggressions. However, information about factors and actions of such PSI was lacking.

Five studies described intervention to reduce behavior-related incidents, such as the adoption of restrictive practices (71), the adoption of unlocked psychiatric wards (i.e., open door policy) (34), and the deployment of specifically trained behavior management teams (72, 73). When it comes to suicides, four studies described interventions such as the use of specifically developed checklists to identify environmental risks (54, 66, 74), and the use of over-the-door alarms (62).

### 3.2 Medication/IV fluids incidents

Nineteen studies described medication-related incidents. Among these, most of the evidence did not specify the PSI detail, while three studies reported the incidence (30, 32, 33). Contributing factors were mainly identified among organizational aspects and staff. Specifically, the lack of adequate policies and procedures has been found contributing to diverse medication-related PSI, such as drug omission or medication dose (55) and to the occurrence of adverse drug reactions (63). When it comes to the staff, inadequate or insufficient communication was identified as a potential risk factor for administration errors (75) and for the occurrence of adverse drug reactions (63). Similarly, the lack of attention during medication processes has been found contributing to incidents related to prescription (76) and the occurrence of adverse drug reactions (63). Actions to reduce risk included provision of monitoring/dispensing equipment and support such as personal digital assistants (77), and the availability of adequate checklists and protocols. No mitigating factors were identified.

Several interventions were described to reduce medication errors. While some authors propose the adoption of technological devices such as personal digital assistants or electronic medical handovers to support all the phases of the medication process (77–79), Kelly et al. stressed the importance of focusing on the patient identification process to reduce the administration of drugs to the wrong patient (80). Contact-free visual monitoring (81) and a nationwide structured training program for professionals (67), were suggested as interventions to prevent self-harm and to reduce the inappropriate use of seclusion and restraints, respectively.

### 3.3 Patient accidents

All the 15 studies dealing with patient accidents described fall events. Of them, six reported the PSI incidence (27–31, 82). The physical environment was identified as one of the most important contributors to such incidents. Indeed, the most common areas of falls were patients’ bedrooms and bathrooms, due to the staff inability to continuously monitor patients (28, 29, 83). Moreover, patient characteristics such as unsteady gait, history of falls, agitation, and comorbidity (84) were identified as potential contributors. Staff poor communication about fall risk in the inpatient setting was identified as other potential contributing factors (55). Actions to reduce risk included the provision of safety equipment (e.g., restraints) (85) and specific patient care (73, 85), but also the improvement of safety culture (85), reorganization of the environment (adoption of the open door policy) (28, 85), and specific staff training (31, 84). Similarly to medication-related incidents, also for patient incidents no potential mitigating factors were described.

An operational intervention plan based on continuous staff education and training was proposed to reduce the occurrence of falls incidence (31).

### 3.4 Clinical processes and procedures incidents

Four studies (53, 61, 86, 87) dealt with clinical process and procedures-related incidents. More specifically, incomplete or inadequate diagnosis (53), care and management (53, 87) were identified as potential PSI. Contributing factors and risk-reduction actions were identified exclusively for incidents related to non-performed treatments, specifically staff unpleasant/aggressive behavior as contributing factor and the fostering of safety culture as mitigation measure (86).

### 3.5 Other patient safety issues

Fourteen studies discussed other patient safety issues. Among them, the identified contributing factors were related to staff and patient (e.g., polytherapy, patient substance use disorders, lack of contact with relatives) (40–42), to the organization and services (e.g., budget constraints, inadequate staffing levels, competing priorities, lack of patient safety and of just culture) (40, 42, 46, 48), and to the physical environment (e.g., absence of de-escalation areas, closed rooms) (46, 48, 65). Identified actions to reduce risk were directed only to leadership improvement (44) and staff orientation (e.g., promoting open discussions on patient safety, and mitigating blame and distance between staff and leaders) (65).

Three studies described actions to reduce risk. Proposed organizational initiatives were the implementation of a specific search protocol to prevent the presence of unsafe items (69), the constitution of experience feedback committees to examine patient safety reports on a monthly basis (27), and the adoption of a patient engagement-based care model (35). In addition, five studies described initiatives to improve safety culture. These included the use of incident reporting systems as a tool with the potential to influence workers awareness and knowledge on patient safety (33, 36, 61), the implementation of a mindfulness program (30), a teamwork-based visual method for healthcare staff to recognize risks and on a daily basis (i.e., “Green Cross Method”) (45).

## 4 Discussion

To the best of the authors’ knowledge, this is the first systematic review examining PSIs within inpatient psychiatric settings using the ICPS framework. Our findings provide a comprehensive, structured, and updated overview of the evidence on PSI, as well as of their contributing factors, actions to reduce risk, and mitigating factors.

In line with previous reviews focused only on safety in psychiatric setting (5), many of the included studies were descriptive or qualitative. Moreover, the limited number of included studies, especially when compared to review conducted in other medical fields, is concerning (88, 89).

Although no research has specifically addressed the lack of quantitative data in psychiatry, several factors likely contribute. Psychiatry traditionally focused on subjective observation of patients rather than quantitative measurement (90), influencing research approaches. Incident reporting systems are underused in psychiatry compared to other medical fields (91). Barriers to reporting, such as a lack of safety culture (92, 93) and mental health stigma (94), may further limit PSI data. Additionally, psychiatric patients could have been marginalized within the medical and scientific communities, which has led to limited research and delayed adoption of safety practices (95). The increased presence of qualitative research (96) likely reflects a growing interest in capturing the complexity of patient experiences and organizational factors in psychiatric settings. This expanded application in psychiatric patient safety research in recent years may have contributed to a relative under-representation of quantitative studies, particularly those providing robust incidence estimates and outcome measures.

Moreover, only few studies reported an estimate of frequency, making the comparison to general hospital setting challenging.

Most of the studies in our review were from the United States and Europe, with little evidence from South America or Africa. Several general patient safety initiatives exist globally (97), with specific programs for these regions (98, 99). This lack of evidence could be due to two factors. First, developing countries are under-represented in research, particularly in psychiatry (100, 101). Second, stigma around mental health issues, especially in Latin America and Africa (102, 103), may hinder the implementation of patient safety initiatives for psychiatric patients.

In line with previous literature, research was mainly conducted in the hospital settings (5). However, the lack of evidence from long-term care (LTC) settings is concerning. Indeed, hospitals represent only 22% of psychiatric beds in Europe (104), with the majority of psychiatric inpatients in community or LTC settings. PSI rates in LTC settings are similar, if not higher, than in acute hospitals (105). Many LTC residents are older, with more than 30% diagnosed with psychiatric disorders, increasing the number of vulnerable individuals at risk for psychiatry-related PSIs.

Moreover, it is worth mentioning that nearly half of the included studies were graded as low or medium quality. Despite, in our review, we did not exclude studies based on quality appraisal results, this aspect is of particular concern.

### 4.1 Patient safety incidents in psychiatry

According to our results, the most frequently identified ITC were Behavior-related incidents, Medication/IV fluids, Patient accidents, and Other patient safety issue. Specifically, Behavior-related incidents were the most frequently reported PSIs, appearing in 56 out of the 88 included studies. Indeed, it has been estimated that more than one third of inpatient psychiatric subjects shows a potentially dangerous behavior (106). In line, self-harm and suicide incidents appear to be eight time more frequent than the general medical setting. On the other hand, such behavior related incidents have been described as extremely common also in the general hospital setting (107). On this regard, a recent systematic review (108) identified some shared strategies to reduce risk, such as staff training, patient education, environmental adjustments, and specifically developed protocols (108). Under this point of view, behavior-related incidents and their preventive strategies in psychiatry and in the general hospital setting could be considered similar, and the translation of knowledge and evidence across the different disciplines should be encouraged.

In line with literature on general care, medication errors are the second most common category of incidents. However, the identified incidence rate was one-fourth compared to the general medical setting. Main common contributing factors were related to staff performance (e.g., inadequate communication, non-consideration of drug interactions), understaffing, and lack of adequate protocols and procedures (109). Some proposed interventions to reduce risk were analogue (i.e., use of electronic/computerized devices, staff training), while the availability of checklists and protocol was described only in psychiatry (110). This issue might be due to the fact that the implementation of protocols and guidelines in psychiatry is particularly complex, with several context-specific barriers (e.g., perceived limited validity of guidelines, lack of organizational skills, and emotional exhaustion) hampering their adoption (111).

The majority of reported PSI were falls, indicating that they constitute the third most common type of PSI in the inpatient psychiatric setting, with an incidence rate in line with the general hospital setting (112). Moreover, we found that actions to reduce this risk were mainly related to environmental changes and staff training. Moreover, despite it has been widely acknowledged that psychotropic drugs significantly increase the risk of falls (113), we did not find any risk reduction strategy aimed to reduce, or at least rationalize, the use of such medications. Indeed, while fall risk is widely acknowledged in psychiatry, this topic is not described, nor managed, as a patient safety issue (114). This evidence corroborates the fact that, especially when compared to other settings, patient safety culture in psychiatry is alarmingly limited (115).

Other ITC reported in the sources, though less frequently as main categories, include Documentation, Resources/organizational management, and Clinical process/procedure. This last category suggests that incidents related to clinical processes and procedures are a critical concern within psychiatric settings, often presenting a higher prevalence compared to general healthcare. Finally, 14 studies discussed other patient safety issues, but without clearly defining specific PSIs.

### 4.2 Contributing factors to PSI in psychiatry

Contributing factors to Patient Safety Incidents (PSI) in psychiatry refer to the underlying elements or circumstances that increase the likelihood of safety incidents occurring within psychiatric settings. These factors can be related to various aspects of care, such as the patients, healthcare staff, the physical environment, and the organizational systems.

Patient factors are listed as a contributing factor heading in the source 18 times across different incident types. This suggests that characteristics and actions specific to the patient are very frequently identified as contributing to safety incidents. Research confirms that adverse social factors exacerbate psychiatric symptoms and reduce engagement with care services, thereby heightening the likelihood of critical incidents. Furthermore, disease state—including the severity and chronicity of psychiatric illness—directly influences the risk profile, as acute phases of illnesses like schizophrenia or bipolar disorder are associated with higher rates of adverse events (116). Importantly, recognizing patient-specific factors as contributors to safety incidents emphasizes the necessity of a proactive, individualized approach to risk management.

Staff factors are also highly frequent contributors. Staff performance, including clinical decision-making and the ability to recognize and respond appropriately to patient deterioration, plays a crucial role in incident prevention. Research by Garrouste-Orgeas et al. (117) demonstrated that staff errors, particularly those arising from cognitive overload, fatigue, or insufficient training, significantly contribute to preventable adverse events in healthcare. The number and quality of staff available is also critical. Studies have shown that insufficient staffing levels or a lack of experienced staff increases the likelihood of safety incidents, particularly in high-risk environments like acute psychiatric wards (118). Recognizing staff-related factors as frequent contributors to safety incidents highlights the need for continuous training, adequate staffing ratios, strong team communication practices, and supportive workplace cultures to ensure both staff and patient safety in psychiatric services.

Organizational/service factors are listed 12 times as a contributing factor category heading issues within the structure, processes, and management of the healthcare service or organization are significant. We found that deficiencies in protocols, policies, and clinical procedures can lead to inconsistencies in care delivery, making it difficult for staff to follow best practices or respond effectively to emergencies. In fact, a study by Flodgren et al. (119) emphasized that the absence of clear, evidence-based guidelines and procedures increases variability in clinical practice, subsequently raising the risk of safety incidents. The organization and coordination of teams are essential. Poor team functioning, lack of clarity in roles, and inadequate inter-professional collaboration can impair clinical decision-making and delay critical interventions. Research has shown that structured team processes, such as multidisciplinary meetings and collaborative care models, significantly enhance safety in psychiatric services (120). Overall, these findings underscore that improving organizational structures, fostering a safety-oriented culture, and ensuring adequate resources and well-defined processes are fundamental strategies for reducing patient safety incidents in psychiatric settings.

The Work/environment factors category appears 9 times as a contributing factor heading, highlighting the role of the physical surroundings and immediate work environment. The primary specific factor listed within this category is Physical environment/infrastructure. Several studies have highlighted the relationship between the built environment and patient safety. For instance, Shepley et al. (121) found that the physical design of psychiatric facilities, including aspects like clear sightlines, reduced opportunities for ligature, and access to calming spaces, directly impacts the rates of violence, suicide attempts, and staff injuries. Inadequate infrastructure, such as poorly designed patient rooms, lack of private spaces, or faulty alarm systems, can delay staff response times during emergencies and hinder therapeutic engagement. Improvements in environmental factors can significantly reduce the frequency and severity of safety incidents and contribute to both patient recovery and staff wellbeing.

### 4.3 Actions to reduce risk of PSI and mitigating factors

Actions to reduce the risk of Patient Safety Incidents (PSIs) refer to the strategies, interventions, and practices implemented within healthcare settings to prevent incidents that could harm patients. When examining **Table 4**, several categories appear more frequently than others. The most frequently appearing main category is Organizational/environmental factors. This category is listed as a heading 14 times across different incident types. Actions within this category focus on the broader system, structure, and physical setting of healthcare. Shepley et al. (121) demonstrated that psychiatric settings designed with safety in mind—featuring ligature-resistant fixtures, clear sightlines, and calming environments—are associated with reduced rates of aggression, self-harm, and absconding. Environmental modifications not only enhance safety but also promote therapeutic engagement and recovery. Organizational culture, particularly the emphasis on a positive safety culture, plays a transformative role in mitigating risks. Mannion and Davies (122) found that a culture which encourages transparency, continuous learning, and non-punitive responses to errors is associated with lower incident rates. Fostering psychological safety among staff encourages early reporting of concerns, near misses, and unsafe conditions, enabling timely interventions. Altogether, these findings underline that preventing patient safety incidents in psychiatry requires not only individual vigilance but also robust organizational frameworks and thoughtfully designed environments that support both staff and patients.

Closely following is the Staff factors category, listed 13 times as a main heading for actions to reduce risk. Actions here are directed at the healthcare workforce. The high frequency of these categories indicates a significant focus in the reviewed studies on addressing staff capabilities and training as primary means of preventing patient safety incidents. Several studies have emphasized that effective, continuous training for healthcare workers significantly reduces the likelihood of adverse events. For instance, Renedo et al. (123) highlighted that strengthening clinical skills, communication abilities, and risk management knowledge among healthcare professionals leads to fewer errors and safer management of complex patient behaviors. Specific training interventions, such as aggression management, verbal de-escalation techniques, and suicide prevention strategies, have proven particularly effective in psychiatric settings. The “Safewards” trial by Bowers et al. (124) demonstrated that targeted staff training significantly reduced incidents of conflict and the need for coercive measures like seclusion and restraint in psychiatric wards.

Mitigating factors of Patient Safety Incidents (PSI) refer to the conditions, actions, or interventions that reduce the severity of an incident or help prevent further harm once a safety incident has occurred. The most frequently described mitigating factors are those “Directed to the organization” and “Directed to staff”. It is notable that the most frequent items also appear under the “Actions to Reduce Risk” column, suggesting that some factors can act both as preventative measures and as mitigators when an incident occurs. Leonard et al. (125) noted that structured communication tools, such as SBAR (Situation-Background-Assessment-Recommendation), not only prevent misunderstandings that lead to patient harm but also support quick, coordinated team responses during crises. This duality underlines the importance of seeing organizational structures and staff competencies not only as static preventive barriers but as active components in dynamic safety management systems, where continuous adaptation, response, and recovery are necessary to maintain patient safety.

Among our included studies, only one-third described interventions to reduce the occurrence of PSI or to increase patient safety. This lack of evidence on interventions is particularly alarming, especially considering literature from other settings. As an example, a review conducted in primary care identified more than one hundred different tools (among the 1300 included studies) to improve patient safety. As it can be hypothesized that some of these interventions might be effectively translated to psychiatry, the fact that none of our included studies adopted such approach corroborates the hypothesis that patient safety in psychiatry could be currently managed and studies separately from other care settings. Therefore, efforts should be directed not only toward filling the gap caused by the lack of experimental studies but also toward translating the body of evidence from other care settings to psychiatry.

### 4.4 Interconnections and relations between the different aspects of patient safety

One of the strengths of our work is the identification of the relation between the different aspects of patient safety in the inpatient psychiatric setting, shown in the Sankey flow diagram (**Figure 3**). Overall, this illustrates the strong interconnections between different PSIs and shows that specific preventive measures can positively impact various incident types. It also confirms that PSIs, particularly in psychiatry, are rarely isolated events triggered by a single cause (5, 126). This is particularly concerning, since most studies included in our review focused on single or few PSIs, whereas our data suggests a systemic approach is necessary to understand patient safety complexity. As noted previously, patient-related incidents were the most common ITCs. Interestingly, the contributing factors for these events were multifactorial, with service organization, staff, and environmental factors contributing just as much as patient-related factors. We observed a similar pattern in actions taken to reduce risk: our evidence highlights a multi-dimensional impact rather than a direct link between a single action and a single factor. In contrast, mitigating factors were mostly related to patient behavior PSIs. While our review highlights the complex and multiple interconnections between the abovementioned aspects, these unique relationships could be likely attributed to a lack of comprehensive evidence, as already reported in literature (6). Under this point of view, the adoption of the ICPS as guiding framework allowed a comprehensive understand of relationships, avoiding a biased approach which could have been introduced when adopting an *a priori*, specifically defined, classification.

**Figure 4:**
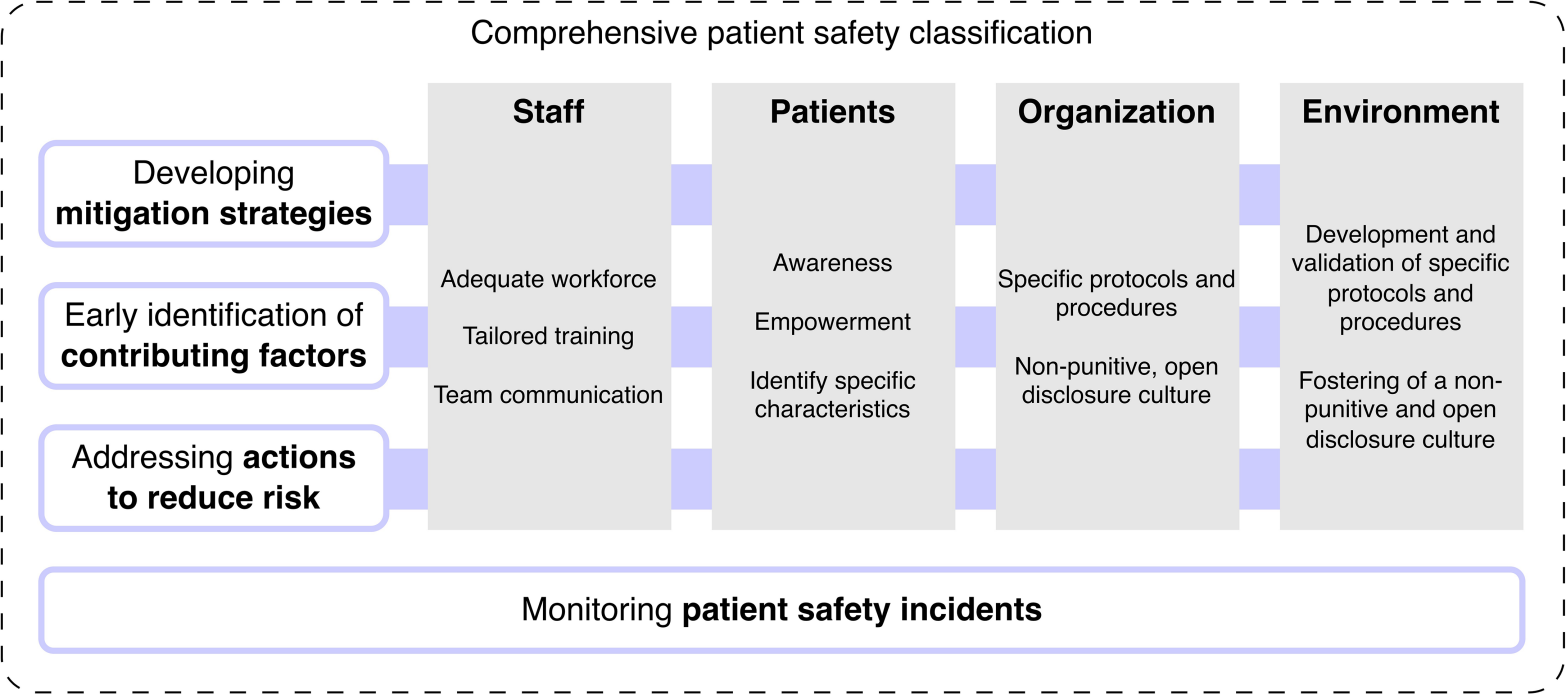
The proposed multi-component and multi-level approach for developing patient safety strategies in psychiatric inpatient settings.

Moreover, the results from our work can inform and support the development of clinical practice guidelines and patient safety initiatives. Indeed, to manage the complexity of PSI in inpatient psychiatric services in an effective and efficient way, we advocate the development of strategies using a multi-component, multi-level approach. According to our results, interventions should be primarily targeted to four domains: i) staff: adequate workforce (e.g., providing an adequate staff number to reduce behavior-related incidents (57)), tailored training (e.g., a nationwide structured training program (67)), and improving team communication (e.g., improving intra-team communication to reduce fall risk (55)); ii) patients: fostering awareness (e.g., promoting incident reporting (33)), empowerment (e.g., providing patients with training and decision support (68)), and identifying specific patient characteristics (e.g., history of alcohol and substance abuse (61)); iii) organization: promotion of a non-punitive and open disclosure culture (e.g., the development of a specific safety culture to reduce involuntary treatments (86)), and specific protocol development and implementation (e.g., protocols for suicide prevention (108)); iv) work environment: availability of adequate devices (e.g., electronic medical handover to support the medication process (78)), and provisioning of a quiet and safe environment (e.g., the implementation of an open door policy (85)). Furthermore, strategies should simultaneously address all the analyzed levels (i.e., actions to reduce risk, early identification of contributing factors, and development of mitigating strategies). This approach should be also informed by the monitoring of patient safety incidents (e.g., using effective incident reporting systems) and addressed adopting a comprehensive and complete patient safety classification, such as the ICPS. This model, shown in **Figure 4**, can support healthcare professionals and policy makers to develop effective interventions to face the complex issue of patient safety in the inpatient setting, and might be also translated to other health sectors.

### 4.5 Limitations

Our study presents some limitations. First, despite search strings were developed to embrace most studies on patient safety in the psychiatric inpatient setting, some studies could have been not captured. As above mentioned, our results are largely based on qualitative or descriptive evidence. Despite this issue could be mainly reconducted to the research trends on this topic, it could have affected our results. Second, our thematic synthesis considered all the included study designs. Despite a more rigorous approach might have given more strength to results, we preferred prioritizing a comprehensive and complete synthesis. Third, despite the ICPS is a general framework developed for all care contexts, it could not be perfectly suitable for inpatient psychiatry. Indeed, the intrinsic complexity of patient safety, together with the complexity of psychiatric patients, might hamper a proper classification, which could miss to capture some aspects.

Finally, the AI-driven approach which we adopted for quality appraisal should be considered as a potential limitation of the current study. Indeed, while this method offered efficiency and consistency, allowing a rapid evaluation of more than 90 original studies, it represents a potential deviation from the original study protocol published on PROSPERO, which did not specify the quality appraisal approach. Moreover, it relies on a technology that is still emerging in the field of systematic reviews, potentially lacking the nuanced interpretive capabilities of a human reviewer, particularly when assessing complex qualitative studies (127). In this regard, it is worth mentioning that our study aimed to provide a complete overview of current knowledge on PSI as well as preventive and mitigation strategies, without making recommendations based on study quality, and results must be interpreted with this methodological novelty in mind. Indeed, while our pilot validation showed a promising adjusted accuracy rate of 85% and higher, we currently cannot suggest the adoption of the same approach for reviews which 1) have a clinical, quantitative outcomes, or 2) exclude studies considering a methodological quality level.

## 5 Conclusion

Our systematic review provides a comprehensive and updated overview of patient safety within inpatient psychiatric settings, adopting the ICPS as guiding framework, as well as of their contributing factors, actions to reduce risk, and mitigating factors. Patient safety within psychiatric inpatient settings remains a critical yet underexplored aspect of healthcare. The predominance of descriptive and qualitative studies highlights a significant gap in quantitative research, as well as a lack of evidence-based interventions tailored to psychiatric care. Additionally, the concentration of research in high-income countries underscores the need for more inclusive and globally representative studies. The lack of evidence from long-term care facilities, where many psychiatric inpatients reside, further emphasizes an urgent area for investigation. To advance patient safety in psychiatric inpatient facilities, comprehensive training programs for staff, the development of tailored protocols, and modifications to the physical environment are imperative. Moreover, fostering a robust safety culture that encourages incident reporting and continuous improvement is essential. Adopting and adapting successful patient safety strategies from other healthcare disciplines could provide immediate and actionable solutions to enhance safety standards in psychiatric settings. Future research should prioritize quantitative studies to evaluate the effectiveness of specific interventions and expand the evidence base across diverse psychiatric care settings globally. By addressing these gaps, healthcare systems can significantly reduce the incidence of PSIs and improve the overall quality of psychiatric inpatient care.

## Data Availability

The original contributions presented in the study are included in the article/**Supplementary Material**. Further inquiries can be directed to the corresponding author.
