# Supplementary material for: Patient safety incidents in the psychiatric inpatient setting: determinants, consequences, and strategies. A systematic review
Source: Front Psychiatry. 2026 Jan 14;16:1703768. doi: 10.3389/fpsyt.2025.1703768 (PMC12848375; doi:10.3389/fpsyt.2025.1703768)
Supplement: Supplementary file 1 [file DataSheet1.pdf]

Patient Safety Incidents in the Psychiatric Inpatient Setting: Determinants, Consequences, and Strategies. A Systematic Review.

Supplementary material

1 Quality appraisal

1.1 Adopted methodology

To perform the AI-based quality appraisal, we used the **GPT-5 Mini**[1] large language model (LLM) developed by OpenAI. This model is the compact variant of the GPT-5 family, developed to balance computational efficiency, latency, and reasoning capability while preserving robust instruction-following and multimodal functionality. Released in August 2025, it shares the same foundation as GPT-5, supporting text and image inputs, structured outputs, tool and function calling, and integration into the Chat and Responses application programming interfaces (API). GPT-5 Mini provides an extended 400,000-token context window for long-form reasoning and dialogue management and is integrated into OpenAI's dynamic routing system, which allocates queries according to task complexity. Initial evaluations highlight its reliability in tool use for diverse applications, making GPT-5 Mini a versatile and efficient model for both research and applied domains.

1.1.1 Script

GPT-5 Mini was accessed using the OpenRouter API. We developed an R script which processed and submitted the manuscript and a specifically-developed prompt to GPT-5 Mini. Results were collected in tabular format (CSV - comma separated values file). It is worth mentioning that we converted the PDF version of the full-text into PNG images, to allow the LLM to perform a comprehensive evaluation which includes also figures, graphs and tables instead of only text. Specifically, the source code (available below) performs the following steps for each full-text PDF:

1. Convert each page of the PDF into an high-resolution PDF image (200 dots per inch);
2. Encodes the images in base64;
3. Sends all the encoded images, togeter with the prompt, to GPT-5 mini via OpenRouter APIs;
4. Retrieves the LLM output and collects it in an R dataframe, with a row for each article;
5. Saves results to a CSV file.

```
# 1. Libraries
library(httr)
library(jsonlite)
library(base64enc)
library(pdftools)

# 2. Config
API_KEY <- "[OPENROUTER API KEY HERE]"
OPENROUTER_API_URL <- "https://openrouter.ai/api/v1/chat/completions"
PDF_FOLDER <- "[FULL-TEXT FOLDER PATH HERE]"
MODEL <- "openai/gpt-5-mini"

output_csv <- paste(PDF_FOLDER, "gpt_qualityappraisal_output.csv", sep = "/")

PROMPT <- "[INSERT PROMPT HERE]"

# 3. PDF to PNG conversion function
convert_pdf_to_images <- function(pdf_path, dpi = 200) {
  message(paste("Convert", pdf_path, "in images..."))
  png_files <- pdf_convert(pdf_path, dpi = dpi)
  return(png_files)
}

# 4. Base64 encoding function
encode_image_to_base64 <- function(file_path) {
  raw_data <- readBin(file_path, "raw", file.info(file_path)$size)
  base64enc::base64encode(raw_data)
}

# 5. Send images to OpenRouter API function
send_images_to_openrouter <- function(images_base64) {
  headers <- add_headers(
    "Authorization" = paste("Bearer", API_KEY),
    "Content-Type" = "application/json"
  )

  content_list <- list(list(type = "text", text = PROMPT))
  for (img64 in images_base64) {
    content_list <- append(content_list, list(
      list(
        type = "image_url",
        image_url = list(url = paste0("data:image/png;base64,", img64))
      )
    ))
  }

```

[1] https://platform.openai.com/docs/models/gpt-5-mini

```r
  messages <- list(list(role = "user", content = content_list))

  body <- list(
    model = MODEL,
    messages = messages
  )

  response <- tryCatch({
    POST(url = OPENROUTER_API_URL,
         body = toJSON(body, auto_unbox = TRUE),
         headers)
  }, error = function(e) {
    message("API ERROR", e$message)
    return(NULL)
  })

  if (is.null(response)) return("API request error")

  result_content <- content(response, "parsed")

  if (!is.null(result_content$choices) &&
      length(result_content$choices) > 0 &&
      !is.null(result_content$choices[[1]]$message$content)) {
    return(result_content$choices[[1]]$message$content)
  } else {
    message("Invalid API response: ",
            jsonlite::toJSON(result_content, auto_unbox = TRUE, pretty = TRUE))
    return("Error: empty or invalid responde.")
  }
}

# 6. Main loop
results <- data.frame(
  "Filename" = character(),
  "GPT_output" = character(),
  stringsAsFactors = FALSE
)

files <- list.files(PDF_FOLDER, pattern = "\\.pdf$", full.names = TRUE)

if (length(files) > 0) {
  for (file_path in files) {
    filename <- basename(file_path)
    message(paste("File:", filename, "..."))

    png_files <- convert_pdf_to_images(file_path, dpi = 200)
    images_base64 <- lapply(png_files, encode_image_to_base64)
    api_response <- send_images_to_openrouter(images_base64)

    new_row <- data.frame(
      "Filename" = filename,
      "GPT_output" = as.character(api_response),
      stringsAsFactors = FALSE
    )
    results <- rbind(results, new_row)
  }

  # 7. Save to CSV
  write.csv(results, file = output_csv, row.names = FALSE)
  message(paste("Output saved in:", output_csv))

} else {
  message("No PDF found in the specified folder.")
}
```

1.1.2 Prompts

The prompts were developed using an iterative approach, in which the content (written following OpenAI guidelines for prompt engineering[2]) was recursively improved using ChatGPT. The specific checklists were obtained from the Joanna Briggs Institute Critical Appraisal Tools webpage[3] and were used in the prompts without any edits. Since the included records were manually classified by the authors according to study design, the script was run once for each study design, with the appropriate checklist included in the prompt.

```
# IDENTITY

I am an academic researcher and you are my research assistant.
You are a professional reviewer with strong expertise in formal quality appraisal of scientific studies.
You learn and follow evaluation criteria precisely, and your judgments are careful, conservative, and unbiased.

# CONTEXT

I am conducting a systematic review and need a quality appraisal of a study.
You will receive the full article as a PDF (pages may be images).

# SCOPE & EVIDENCE RULES

-   Use ONLY the content inside the provided article (including title page, abstract, main text, figures, tables,
    appendices, and footnotes if present). Do not use external knowledge.
```

[2] https://platform.openai.com/docs/guides/prompt-engineering
[3] https://jbi.global/critical-appraisal-tools

```
-   If the PDF is image-based or low-quality, infer only what is clearly legible;

# METADATA EXTRACTION (for table columns 1-3)

-   First Author Name: Use the surname of the first listed individual author exactly as written; if only a
   group/consortium is listed, use that name; if truly absent, write UNKNOWN.
-   Publishing Year: Use the final publication year (journal citation). If unavailable, use the year stated on the
   title page; if none, write NA.
-   DOI: Output only the bare DOI string (e.g., 10.1234/abcd.efgh). Remove any https. If no DOI present, write NA.

# JUDGMENT SCALE (strict)

-   YES: The criterion is clearly satisfied. Minor, non-impacting gaps may exist but do not undermine clarity or
   appropriateness.
-   NO: The criterion is inadequately addressed or contradicted; major gaps undermine clarity or appropriateness.
-   UNCLEAR: Some indication exists, but information is insufficient, ambiguous, or not specific enough to decide
   YES or NO.
-   NA: The criterion does not apply to this study due to: population characteristics, study design, investigated
   topic.

# CHECKLIST

Below you find the checklist (list of questions).
Each question is numbered, and specific details about each question are listed between square brackets.

>>> Below, an example of questions. Replace with appropriate questions from JBI checklists. <<<<

1.  Is there congruity between the stated philosophical perspective and the research methodology?
    [Does the report clearly state the philosophical or theoretical premises on which the study is based?
    Does the report clearly state the methodological approach adopted on which the study is based?
    Is there congruence between the two?
    For example: A report may state that the study adopted a critical perspective and participatory action research
       methodology was followed.
    Here there is congruence between a critical view (focusing on knowledge arising out of critique, action and
       reflection) and action research (an approach that focuses on firstly working with groups to reflect on
       issues or practices, then considering how they could be different; then acting to create a change; and
       finally identifying new knowledge arising out of the action taken).
    However, a report may state that the study adopted an interpretive perspective and used survey methodology.
    Here there is incongruence between an interpretive view (focusing on knowledge arising out of studying what
       phenomena mean to individuals or groups) and surveys (an approach that focuses on asking standard questions
       to a defined study population); a report may state that the study was qualitative or used qualitative
       methodology (such statements do not demonstrate rigour in design) or make no statement on philosophical
       orientation or methodology.]


# TIE-BREAK & CONSERVATIVE RULES

-   When evidence is borderline between YES and UNCLEAR, choose UNCLEAR.
-   When evidence is borderline between NO and UNCLEAR, choose NO only if contradictions or major gaps are
   explicit; otherwise UNCLEAR.
-   Do not infer beyond text; absence of evidence is not evidence of presence.

# OUTPUT FORMAT (STRICT)

-   Produce ONLY a Markdown table with NO header row.
-   Column order EXACTLY: First Author Name \| Publishing Year \| DOI \| Q1 \| Q2 \| Q3 \| Q4 \| Q5 \| Q6 \| Q7 \|
   Q8 \| Q9 \| Q10
-   Each cell is a single token from {YES, NO, UNCLEAR, NA} for Q1-Q10.
-   No extra commentary, notes, citations, or explanations.
-   Examples of valid rows (do not fabricate; these are format examples only, not to be output if not applicable):
   \| Smith \| 2021 \| 10.1000/xyz123 \| YES \| YES \| UNCLEAR \| YES \| YES \| NO \| UNCLEAR \| YES \| YES \| YES
   \|

# PROCESS (INTERNAL, DO NOT OUTPUT)

1)  Read the entire article; 2) Extract metadata (author/year/DOI) per rules; 3) For each item, apply the
   heuristics above; 4) Resolve ties using TIE-BREAK rules; 5) Output the table only.
```

1.1.3 Validation

To check the reliability of automated AI screening, we evaluated the LLM outputs. Specifically, 15% of the included records (randomly selected) for each study design were also manually appraised by a human researcher, and results were compared to those obtained from the LLM. In case of disagreement between the LLM and the human researcher, a second researcher provided the final evaluation to assess the final agreement level. It is worth mentioning that study designs for which there was only one study each (i.e., case-control, Delphi, and case report studies) were manually appraisaed without using LLM. Therefore, we calculated raw and adjusted agreement ratios. Specifically, we defined:

- I_c Items for which both human and LLM reviewers agreed (both "yes", "no", "N/A", or "unclear");
- I_u Items for which one reviewer (human or LLM) answered yes, while the other answered no;
- I_p Items for which one reviewer answered "yes" or "no" and the other reviewer answered "unclear";
- $f(x)$ The number of times each variable is present in the sample (frequency).

The **raw agreement rate** is defined as:

$$A_r = \frac{f(I_c)}{f(I_c) + f(I_u) + f(I_p)} \tag{1}$$

and the **adjusted agreement** is defined as:

$$A_a = \frac{f(I_c) + 0.5f(I_p)}{f(I_c) + f(I_u) + f(I_p)} \tag{2}$$

The following table shows the final agreement level, per study design.

Study design	A_r	A_a
Qualitative studies	80%	93%
Cross-sectional studies	88%	92%
Mixed-methods studies	100%	100%
Narrative evidence	81%	85%
Cohort studies	81 %	90%
Prevalence studies	100%	100%
Quasi-experimental	83%	88%
Case series	100%	100%

1.2 Qualitative studies

First author	Year	1	2	3	4	5	6	7	8	9	10	Overall
Adler	2020	Y	Y	Y	Y	Y	Y	Y	Y	Y	Y	100%
Anderson	2013	Y	Y	Y	Y	Y	U	U	Y	Y	Y	80%
Asikainen	2020	U	Y	Y	Y	Y	U	U	N	Y	Y	60%
Berzins	2020	Y	Y	Y	Y	Y	U	Y	Y	Y	Y	90%
Derblom	2021	U	Y	Y	Y	Y	Y	Y	Y	U	Y	80%
Digby	2020	U	Y	Y	Y	Y	U	Y	Y	Y	Y	80%
Duxbury	2010	N	Y	Y	Y	Y	N	Y	Y	Y	Y	80%
Jayaram	2011	NA	Y	Y	Y	Y	NA	NA	NA	N	Y	83%
Kelly	2011	N	Y	Y	Y	Y	Y	U	Y	Y	Y	80%
Koukia	2009	N	Y	Y	Y	Y	N	N	Y	Y	Y	70%
Kuosmanen	2017	U	Y	Y	Y	Y	N	N	Y	Y	Y	70%
Kwobah	2023	N	Y	Y	Y	Y	N	N	Y	Y	Y	70%
Lessard-Deschênes	2022	Y	Y	Y	Y	Y	Y	U	Y	Y	Y	90%
Lindwall	2011	Y	Y	Y	Y	Y	Y	Y	Y	Y	Y	100%
Mitchell	2001	Y	Y	Y	Y	Y	Y	Y	Y	Y	Y	100%
Olasoji	2024	U	Y	Y	Y	Y	N	N	Y	Y	Y	70%
Powell-Cope	2014	Y	Y	Y	Y	Y	N	Y	Y	Y	Y	90%
Price	2024	Y	Y	Y	Y	Y	Y	Y	Y	Y	Y	100%
Maddineshat	2024	N	Y	Y	Y	Y	N	Y	Y	N	Y	70%
True	2017	Y	Y	Y	Y	Y	N	N	Y	Y	Y	80%
Vahidi	2018	U	Y	Y	Y	Y	N	Y	Y	Y	Y	80%
Vermeulen	2019	Y	Y	Y	Y	Y	N	Y	Y	Y	Y	90%
Zola	2024	Y	Y	Y	Y	Y	U	N	Y	Y	Y	80%

Questions

1. Is there congruity between the stated philosophical perspective and the research methodology? parallel groups) accounted for in the conduct and analysis of the trial?
2. Is there congruity between the research methodology and the research question or objectives?
3. Is there congruity between the research methodology and the methods used to collect data?
4. Is there congruity between the research methodology and the representation and analysis of data?
5. Is there congruity between the research methodology and the interpretation of results?
6. Is there a statement locating the researcher culturally or theoretically?
7. Is the influence of the researcher on the research, and vice-versa, addressed?
8. Are participants, and their voices, adequately represented?
9. Is the research ethical according to current criteria or, for recent studies, and is there evidence of ethical approval by an appropriate body?
10. Do the conclusions drawn in the research report flow from the analysis, or interpretation, of the data?

Reference Lockwood C, Munn Z, Porritt K. *Qualitative research synthesis.* International Journal of Evidence-Based Healthcare. 2015;13(3):179-187.

1.3 Analytical cross-sectional studies

First author	Year	1	2	3	4	5	6	7	8	Overall
Chen	2024	Y	Y	Y	Y	Y	Y	Y	Y	100%
Cook	2020	U	Y	U	Y	Y	Y	U	Y	63%
Dückers	2019	Y	Y	Y	Y	Y	Y	Y	Y	100%
Instefjord	2014	Y	Y	U	Y	N	N	Y	Y	63%
Källman	2022	Y	Y	Y	Y	Y	U	Y	U	88%
Keers	2015	Y	Y	Y	Y	Y	Y	Y	Y	100%
Kuusisto	2022	Y	Y	U	Y	Y	N	Y	Y	75%
Okkenhaug	2019	U	Y	Y	Y	Y	Y	Y	U	75%
Park	2023	Y	Y	Y	Y	Y	Y	Y	Y	100%
Renwick	2018	Y	Y	U	Y	Y	Y	Y	Y	88%
Sawamura	2005	Y	Y	N	Y	Y	Y	U	Y	75%
Wurst	2013	U	Y	N	Y	U	N	Y	Y	50%

Questions

1. Were the criteria for inclusion in the sample clearly defined?
2. Were the study subjects and the setting described in detail?
3. Was the exposure measured in a valid and reliable way?
4. Were objective, standard criteria used for measurement of the condition?
5. Were confounding factors identified?
6. Were strategies to deal with confounding factors stated?
7. Were the outcomes measured in a valid and reliable way?
8. Was appropriate statistical analysis used?

Reference Joanna Briggs Institute, 2020. *Critical Appraisal Checklist for Analytical Cross Sectional Studies.*

Table S3. Mixed-methods studies

First author	Year	1	2	3	4	5	Overall
Alshehri	2021	Y	Y	Y	Y	Y	100%
Bayramzadeh	2016	Y	Y	Y	Y	Y	100%
Deringer	2014	N	Y	Y	Y	U	60%
Ndebele	2024	Y	U	U	Y	Y	60%
Pelto-Piri	2020	Y	Y	Y	Y	Y	100%
Skelton	2019	U	Y	Y	Y	Y	80%
Varpula	2023	Y	Y	Y	Y	Y	100%
Vruwink	2022	Y	Y	Y	Y	Y	100%

Questions

1. Is there an adequate rationale for using a mixed methods design to address the research question?
2. Are the different components of the study effectively integrated to answer the research question?
3. Are the outputs of the integration of qualitative and quantitative components adequately interpreted?
4. Are divergences and inconsistencies between quantitative and qualitative results adequately addressed?
5. Do the different components of the study adhere to the quality criteria of each tradition of the methods involved?

Reference Hong, Q.N.; Fàbregues, S.; Bartlett, G.; Boardman, F.; Cargo, M.; Dagenais, P.; Gagnon, M.-P.; Griffiths, F.; Nicolau, B.; O'Cathain, A.; et al. The Mixed Methods Appraisal Tool (MMAT) Version 2018 for Information Professionals and Researchers. *EFI* **2018,** 34, 285–291.

1.4 Narrative evidence studies

First Author	Year	1	2	3	4	5	6	Overall
Cardone	2009	Y	Y	Y	Y	Y	Y	100%
Grasso	2001	Y	Y	Y	Y	Y	Y	100%
Marcus	2018	Y	Y	Y	Y	Y	Y	100%

Questions

1. Is the generator of the narrative a credible or appropriate source?
2. Is the relationship between the text and its context explained?
3. Does the narrative present the events using a logical sequence so the reader or listener can understand how it unfolds?
4. Do you, as reader or listener of the narrative, arrive at similar conclusions to those drawn by the narrator?
5. Do the conclusions flow from the narrative account?
6. Do you consider this account to be a narrative?

Reference McArthur A, Klugarova J, Yan H, Florescu S. *Chapter 4: Systematic reviews of text and opinion.* In: Aromataris E, Munn Z (Editors). JBI Manual for Evidence Synthesis. JBI, 2020

1.5 Cohort studies

First Author	Year	1	2	3	4	5	6	7	8	9	10	11	Overall
Chen	2012	Y	Y	U	Y	Y	N	Y	U	U	N	Y	55%
Costopoulos	2019	Y	Y	U	Y	Y	U	Y	Y	Y	Y	Y	82%
Krvavac	2023	Y	Y	Y	Y	N	NA	Y	NA	NA	NA	Y	86%
Riblet	2019	NA	NA	NA	Y	N	NA	Y	Y	N	N	Y	57%

Questions

1. Were the two groups similar and recruited from the same population?
2. Were the exposures measured similarly to assign people to both exposed and unexposed groups?
3. Was the exposure measured in a valid and reliable way?
4. Were confounding factors identified?
5. Were strategies to deal with confounding factors stated?
6. Were the groups/participants free of the outcome at the start of the study (or at the moment of exposure)?
7. Were the outcomes measured in a valid and reliable way?
8. Was the follow up time reported and sufficient to be long enough for outcomes to occur?
9. Was follow up complete, and if not, were the reasons to loss to follow up described and explored?
10. Were strategies to address incomplete follow up utilized?
11. Was appropriate statistical analysis used?

Reference Joanna Briggs Institute, 2020. *Critical Appraisal Checklist for Cohort Studies.*

1.6 Prevalence studies

First Author	Year	1	2	3	4	5	6	7	8	9	Overall
Abraham	2016	Y	Y	U	Y	U	Y	Y	Y	N	67%
Alenzi	2024	Y	Y	Y	Y	Y	Y	Y	Y	NA	100%
Biswas	2023	Y	Y	Y	Y	Y	Y	Y	Y	NA	100%
Boussat	2015	N	Y	Y	Y	Y	Y	U	Y	N	67%
Brooker	2024	Y	Y	Y	Y	N	U	N	N	Y	56%
Glantz	2019	N	N	N	Y	U	Y	Y	U	N	33%
Ito	2003	N	N	U	Y	N	Y	U	Y	N	33%
Keers	2014	Y	Y	Y	Y	U	Y	Y	Y	NA	88%
Lebas	2024	Y	Y	Y	Y	U	Y	Y	U	N	67%
Soerensen	2013	U	N	N	Y	Y	Y	Y	Y	NA	63%
Marcus	2018	Y	Y	Y	Y	Y	Y	Y	Y	Y	100%
Mills	2013	Y	Y	Y	N	Y	Y	Y	Y	NA	88%
Norris	2021	Y	Y	U	Y	U	Y	Y	Y	N	67%
Olashore	2018	Y	Y	Y	Y	Y	Y	Y	Y	Y	100%
Raboch	2010	N	Y	Y	Y	N	Y	Y	Y	N	67%
Reilly	2018	Y	Y	Y	Y	Y	Y	Y	Y	NA	100%
Rothschild	2007	Y	Y	Y	Y	U	Y	Y	Y	NA	88%
Russotto	2024	Y	Y	Y	Y	Y	Y	Y	Y	Y	100%
Schwappach	2019	N	Y	U	Y	N	Y	Y	Y	N	56%
Vargas	2023	Y	Y	N	Y	Y	Y	Y	U	U	67%
Xu	2022	N	N	Y	Y	Y	Y	Y	Y	U	67%

Questions

1. Was the sample frame appropriate to address the target population?
2. Were study participants recruited in an appropriate way?
3. Was the sample size adequate?
4. Were the study subjects and setting described in detail?
5. Was data analysis conducted with sufficient coverage of the identified sample?
6. Were valid methods used for the identification of the condition?
7. Was the condition measured in a standard, reliable way for all participants?
8. Was there appropriate statistical analysis?
9. Was the response rate adequate, and if not, was the low response rate managed appropriately?

Reference Munn Z, Moola S, Lisy K, Riitano D, Tufanaru C. *Chapter 5: Systematic reviews of prevalence and incidence.* In: Aromataris E, Munn Z (Editors). JBI Manual for Evidence Synthesis. JBI, 2020

1.7 Quasi-experimental studies

First Author	Year	1	2	3	4	5	6	7	8	9	Overall
Abela-Dimech	2017	Y	N	Y	U	Y	Y	U	NA	Y	63%

Table 8:

First Author	Year	1	2	3	4	5	6	7	8	9	Overall
Beaglehole	2017	Y	N	Y	N	Y	Y	Y	Y	Y	78%
Brady	2011	Y	N	Y	Y	Y	Y	Y	N	Y	78%
Harrington	2019	Y	N	N	U	Y	Y	U	Y	Y	56%
Kracher	2020	Y	N	U	U	Y	Y	Y	U	N	56%
Kuosmanen	2019	Y	Y	Y	Y	Y	Y	Y	Y	Y	100%
Mills	2010	Y	N	Y	Y	N	Y	U	Y	Y	67%
Mills	2020	Y	N	U	U	N	Y	U	NA	Y	50%
Quigley	2014	Y	N	Y	U	Y	Y	Y	Y	U	67%
Shah	2022	Y	N	U	U	Y	N	U	U	Y	33%
Watts	2012	Y	Y	U	N	Y	N	Y	Y	Y	67%
Watts	2017	Y	N	U	U	Y	Y	Y	N	Y	55%
Whitecross	2020	Y	N	Y	N	Y	Y	U	NA	Y	63%

Questions

1. It is clear in the study what is the “cause” and what is the “effect” (ie, there is no confusion about which variable comes first)?
2. Was there a control group?
3. Were participants included in any comparisons similar?
4. Were the participants included in any comparisons receiving similar
5. treatment/care, other than the exposure or intervention of interest?
6. Were there multiple measurements of the outcome, both pre and post the intervention/exposure?
7. Were the outcomes of participants included in any comparisons measured in the same way?
8. Were outcomes measured in a reliable way?
9. Was follow-up complete and if not, were differences between groups in terms of their follow-up adequately described and analyzed?
10. Was appropriate statistical analysis used?

Reference Barker TH, Habibi N, Aromataris E, et al. *The revised JBI critical appraisal tool for the assessment of risk of bias for quasi-experimental studies.* JBI Evid Synth. 2024;22(3):378-388. Published 2024 Mar 1. doi:10.11124/JBIES-23-00268

1.8 Delphi studies

First Author	Year	1	2	3	4	5	6	Overall
Mascherek	2016	Y	Y	Y	Y	Y	Y	100%

Questions

1. Is the source of the opinion clearly identified?
2. Does the source of the opinion have standing in the field of expertise?
3. Are the interests of the relevant population the central focus of the opinion?
4. Does the opinion demonstrate a logically defended argument to support the conclusions drawn?
5. Is there reference to the extant literature?
6. Is any incongruence with the literature/sources logically defended?

Reference McArthur A, Klugarova J, Yan H, Florescu S. *Chapter 4: Systematic reviews of text and opinion.* In: Aromataris E, Munn Z (Editors). JBI Manual for Evidence Synthesis. JBI, 2020

1.9 Case report studies

First Author	Year	1	2	3	4	5	6	7	8	Overall
Russ	2017	NA	NA	N	NA	Y	NA	Y	Y	75%

Questions

1. Were patient’s demographic characteristics clearly described?
2. Was the patient’s history clearly described and presented as a timeline?
3. Was the current clinical condition of the patient on presentation clearly described?
4. Were diagnostic tests or methods and the results clearly described?
5. Was the intervention(s) or treatment procedure(s) clearly described?
6. Was the post-intervention clinical condition clearly described?
7. Were adverse events (harms) or unanticipated events identified and described?
8. Does the case report provide takeaway lessons?

Reference Joanna Briggs Institute. *Critical Appraisal Checklist for Case Reports.*

1.10 Case series

First Author	Year	1	2	3	4	5	6	7	8	9	10	Overall
Bayramzadeh	2018	Y	Y	Y	Y	Y	Y	U	Y	Y	Y	90%

Table 11:

First Author	Year	1	2	3	4	5	6	7	8	9	10	Overall
Lee	2012	Y	Y	Y	Y	Y	N	N	Y	N	Y	70%
Mills	2017	Y	U	Y	Y	Y	N	N	Y	Y	Y	70%
Raveendranathan	2012	Y	Y	Y	Y	Y	Y	Y	Y	Y	Y	100%
Roos af Hjelmsäter	2019	Y	Y	U	Y	Y	Y	Y	Y	Y	Y	90%

Questions

1. Were there clear criteria for inclusion in the case series?
2. Was the condition measured in a standard, reliable way for all participants included in the case series?
3. Were valid methods used for identification of the condition for all participants included in the case series?
4. Did the case series have consecutive inclusion of participants?
5. Did the case series have complete inclusion of participants?
6. Was there clear reporting of the demographics of the participants in the study?
7. Was there clear reporting of clinical information of the participants?
8. Were the outcomes or follow-up results of cases clearly reported?
9. Was there clear reporting of the presenting site(s)/clinic(s) demographic information?
10. Was statistical analysis appropriate?

Reference Joanna Briggs Institute. *Critical Appraisal Checklist for Case Series.*

1.11 Case-control studies

First Author	Year	1	2	3	4	5	6	7	8	9	10	Overall
Seeherunwong	2022	Y	Y	Y	NA	Y	Y	Y	Y	Y	Y	100%

Questions

1. Were the groups comparable other than the presence of disease in cases or the absence of disease in controls?
2. Were cases and controls matched appropriately?
3. Were the same criteria used for identification of cases and controls?
4. Was exposure measured in a standard, valid and reliable way?
5. Was exposure measured in the same way for cases and controls?
6. Were confounding factors identified?
7. Were strategies to deal with confounding factors stated?
8. Were outcomes assessed in a standard, valid and reliable way for cases and controls?
9. Was the exposure period of interest long enough to be meaningful?
10. Was appropriate statistical analysis used?

Reference Joanna Briggs Institute. *Critical Appraisal Checklist for Case-Control Studies.*

2 PRISMA Checklists

2.1 Manuscript checklist

Section and Topic	Item	Checklist item	Page
**Title**			
Title	1	Identify the report as a systematic review.	1
**Abstract**			
Abstract	2	See the PRISMA 2020 for Abstracts Checklist.	2
**Introduction**			
Rationale	3	Describe the rationale for the review in the context of existing knowledge.	3
Objectives	4	Provide an explicit statement of the objective(s) or question(s) the review addresses.	3
**Methods**			
Eligibility Criteria	5	Specify the inclusion and exclusion criteria for the review and how studies were grouped for the syntheses	4
Information sources	6	Specify all databases, registers, websites, organisations, reference lists and other sources searched or consulted to identify studies. Specify the date when each source was last searched or consulted.	4
Search strategy	7	Present the full search strategies for all databases, registers and websites, including any filters and limits used.	4
Selection process	8	Specify the methods used to decide whether a study met the inclusion criteria of the review, including how many reviewers screened each record and each report retrieved, whether they worked independently, and if applicable, details of automation tools used in the process.	4
Data collection process	9	Specify the methods used to collect data from reports, including how many reviewers collected data from each report, whether they worked independently, any processes for obtaining or confirming data from study investigators, and if applicable, details of automation tools used in the process.	4
Data items	10a	List and define all outcomes for which data were sought. Specify whether all results that were compatible with each outcome domain in each study were sought (e.g. for all measures, time points, analyses), and if not, the methods used to decide which results to collect.	4-5
	10b	List and define all other variables for which data were sought (e.g. participant and intervention characteristics, funding sources). Describe any assumptions made about any missing or unclear information.	4-5
Study risk of bias assessment	11	Specify the methods used to assess risk of bias in the included studies, including details of the tool(s) used, how many reviewers assessed each study and whether they worked independently, and if applicable, details of automation tools used in the process.	4
Effect measures	12	Specify for each outcome the effect measure(s) (e.g. risk ratio, mean difference) used in the synthesis or presentation of results	N/A
Synthesis methods	13a	Describe the processes used to decide which studies were eligible for each synthesis (e.g. tabulating the study intervention characteristics and comparing against the planned groups for each synthesis (item #5)).	5
	13b	Describe any methods required to prepare the data for presentation or synthesis, such as handling of missing summary statistics, or data conversions.	
	13c	Describe any methods used to tabulate or visually display results of individual studies and syntheses.	5
	13d	Describe any methods used to synthesize results and provide a rationale for the choice(s). If meta-analysis was performed, describe the model(s), method(s) to identify the presence and extent of statistical heterogeneity, and software package(s) used.	5
	13e	Describe any methods used to explore possible causes of heterogeneity among study results (e.g. subgroup analysis, meta-regression).	N/A
	13f	Describe any sensitivity analyses conducted to assess robustness of the synthesized results.	N/A
Reporting bias assessment	14	Describe any methods used to assess risk of bias due to missing results in a synthesis (arising from reporting biases).	N/A
Certainty assessment	15	Describe any methods used to assess certainty (or confidence) in the body of evidence for an outcome.	N/A
**Results**			
Study selection	16a	Describe the results of the search and selection process, from the number of records identified in the search to the number of studies included in the review, ideally using a flow diagram.	5
	16b	Cite studies that might appear to meet the inclusion criteria, but which were excluded, and explain why they were excluded.	5
Study Characteristics	17	Cite each included study and present its characteristics.	5
Risk of bias in studies	18	Present assessments of risk of bias for each included study.	5
Results of individual studies	19	For all outcomes, present, for each study: (a) summary statistics for each group (where appropriate) and (b) an effect estimate and its precision (e.g. confidence/credible interval), ideally using structured tables or plots.	6
Results of syntheses	20a	For each synthesis, briefly summarise the characteristics and risk of bias among contributing studies	5-9
	20b	Present results of all statistical syntheses conducted. If meta-analysis was done, present for each the summary estimate and its precision (e.g. confidence/credible interval) and measures of statistical heterogeneity. If comparing groups, describe the direction of the effect.	5
	20c	Present results of all investigations of possible causes of heterogeneity among study results.	N/A
	20d	Present results of all sensitivity analyses conducted to assess the robustness of the synthesized results.	N/A
Reporting biases	21	Present assessments of risk of bias due to missing results (arising from reporting biases) for each synthesis assessed.	N/A
Certainty of evidence	22	Present assessments of certainty (or confidence) in the body of evidence for each outcome assessed.	N/A
**Discussion**			
Discussion	23a	Provide a general interpretation of the results in the context of other evidence.	9-13
	23b	Discuss any limitations of the evidence included in the review.	14
	23c	Discuss any limitations of the review processes used.	14
	23d	Discuss implications of the results for practice, policy, and future research.	14
**Other information**			
Registration and protocol	24a	Provide registration information for the review, including register name and registration number, or state that the review was not registered.	4
	24b	Indicate where the review protocol can be accessed, or state that a protocol was not prepared.	4
	24c	Describe and explain any amendments to information provided at registration or in the protocol.	N/A
Support	25	Describe sources of financial or non-financial support for the review, and the role of the funders or sponsors in the review.	45
Competing interests	26	Declare any competing interests of review authors.	45
Availability of data, code and other materials	27	Report which of the following are publicly available and where they can be found: template data collection forms; data extracted from included studies; data used for all analyses; analytic code; any other materials used in the review.	Tables 1, 2, 3, and 4

2.2 Abstract checklist

Section and Topic	Item	Checklist item	Reported
**Title**			
Title	1	Identify the report as a systematic review.	Yes
**Background**			
Objectives	2	Provide an explicit statement of the main objective(s) or question(s) the review addresses.	Yes
**Methods**			
Eligibility criteria	3	Specify the inclusion and exclusion criteria for the review.	Yes
Information sources	4	Specify the information sources (e.g. databases, registers) used to identify studies and the date when each was last searched.	Yes
Risk of bias	5	Specify the methods used to assess risk of bias in the included studies.	Yes
Synthesis of results	6	Specify the methods used to present and synthesise results.	Yes
**Results**			
Included studies	7	Give the total number of included studies and participants and summarise relevant characteristics of studies.	Yes
Synthesis of results	8	Present results for main outcomes, preferably indicating the number of included studies and participants for each. If meta-analysis was done, report the summary estimate and confidence/credible interval. If comparing groups, indicate the direction of the effect (i.e. which group is favoured).	Yes
**Discussion**			
Limitations of evidence	9	Provide a brief summary of the limitations of the evidence included in the review (e.g. study risk of bias, inconsistency and imprecision).	Yes
Interpretation	10	Provide a general interpretation of the results and important implications.	Yes
**Other**			
Funding	11	Specify the primary source of funding for the review.	Yes
Registration	12	Provide the register name and registration number.	Yes
